# Chronic motor performance following different traumatic brain injury severity—A systematic review

**DOI:** 10.3389/fneur.2023.1180353

**Published:** 2023-05-11

**Authors:** Frances Corrigan, Ing Chee Wee, Lyndsey E. Collins-Praino

**Affiliations:** ^1^Head Injury Lab, School of Biomedicine, The University of Adelaide, Adelaide, SA, Australia; ^2^Cognition, Ageing and Neurodegenerative Disease Laboratory, School of Biomedicine, The University of Adelaide, Adelaide, SA, Australia

**Keywords:** traumatic brain injury, neurodegenerative movement disorder, motor performance, long-term outcomes, systematic review

## Abstract

**Introduction:**

Traumatic brain injury (TBI) is now known to be a chronic disease, causing ongoing neurodegeneration and linked to increased risk of neurodegenerative motor diseases, such as Parkinson's disease and amyotrophic lateral sclerosis. While the presentation of motor deficits acutely following traumatic brain injury is well-documented, however, less is known about how these evolve in the long-term post-injury, or how the initial severity of injury affects these outcomes. The purpose of this review, therefore, was to examine objective assessment of chronic motor impairment across the spectrum of TBI in both preclinical and clinical models.

**Methods:**

PubMed, Embase, Scopus, and PsycINFO databases were searched with a search strategy containing key search terms for TBI and motor function. Original research articles reporting chronic motor outcomes with a clearly defined TBI severity (mild, repeated mild, moderate, moderate–severe, and severe) in an adult population were included.

**Results:**

A total of 97 studies met the inclusion criteria, incorporating 62 preclinical and 35 clinical studies. Motor domains examined included neuroscore, gait, fine-motor, balance, and locomotion for preclinical studies and neuroscore, fine-motor, posture, and gait for clinical studies. There was little consensus among the articles presented, with extensive differences both in assessment methodology of the tests and parameters reported. In general, an effect of severity was seen, with more severe injury leading to persistent motor deficits, although subtle fine motor deficits were also seen clinically following repeated injury. Only six clinical studies investigated motor outcomes beyond 10 years post-injury and two preclinical studies to 18–24 months post-injury, and, as such, the interaction between a previous TBI and aging on motor performance is yet to be comprehensively examined.

**Conclusion:**

Further research is required to establish standardized motor assessment procedures to fully characterize chronic motor impairment across the spectrum of TBI with comprehensive outcomes and consistent protocols. Longitudinal studies investigating the same cohort over time are also a key for understanding the interaction between TBI and aging. This is particularly critical, given the risk of neurodegenerative motor disease development following TBI.

## 1. Introduction

Although once thought of as an acute event, it is now well recognized that traumatic brain injury (TBI) leads to long-lasting disability in a subset of individuals (1–3), including persistent impairments in memory, decision-making, and motor function. Following even mild TBI, 53% of individuals still report functional limitations at 12 months post-injury (4). Such impairments significantly impact an individual's quality of life, affecting social relationships and ability to return to work (5). Mobility, in particular, has been shown to be an important mediator of the relationship between TBI and quality of life following injury (6) with more functional impairment associated with decreases in life satisfaction (7).

Acutely, TBI leads to several neuromotor deficits which are injury severity dependent. Mild TBI most commonly presents with balance disturbance and poor coordination, (8, 9) while severe TBI can lead to spastic paralysis, impaired motor coordination with postural instability and gait abnormalities, and reduced fine motor control (10). Motor impairment has been particularly well-characterized to occur following moderate–severe TBI, with nearly 78% of individuals reporting some level of impairment on gross neuromotor examination during acute rehabilitation (10). Studies focused on the 1^st^ year post-injury in moderate–severe TBI have shown that most motor recovery is reached within 6 months post-injury (11–13), with patients not showing significant functional improvement over the latter part of the year (12, 14, 15). In line with this, 30% of individuals reported difficulty in walking unaided up to 2 years following moderate–severe injury (16), with 25% of individuals still reporting upper- or lower-limb motor difficulty and 43% reporting balance difficulties, even 4 years after a severe brain injury (17). Conversely, following a mild TBI, impairments generally resolve within days to weeks post-injury, although some level of motor dysfunction may persist in at least a subset of individuals [see Chmieliewski et al. for review (18)]. In support of this, slowed motor execution speed and impaired postural control have been reported up to 9 months following concussion in university football players, compared to healthy, non-concussed controls (19).

Despite evidence that motor impairment may persist chronically following TBI, however, examination of the evolution of specific motor deficits long-term following TBI has received comparatively little attention in the literature. Indeed, particularly in clinical research, published TBI outcome studies are skewed toward global measures and/or measures within the behavioral and cognitive, rather than physical, domains. In addition, of studies that do report physical outcomes, most utilize gross functional or disability instruments, rather than dissecting specific types of motor impairment. For example, utilizing the Rivermead Concussion scale, Theadom found 28.5% of participants reporting dizziness at 12 months following mild TBI, (20) which is in line with an earlier Ponsford et al. study, where, on structured interview 2 years post-TBI, 36% of patients reported dizziness (21). Studies where specific motor impairments are reported typically examine only one motor domain; for example, Williams et al. examined chronic gait dysfunction following severe TBI (22–24) and Pearce et al. the effects of prior concussion on fine motor performance (25, 26). Even in preclinical studies, the behavioral batteries employed typically only consist of 1–2 motor specific tasks (27–36) and, thus, cannot provide a comprehensive overview on how TBI influences motor performance as a whole.

Understanding the persistent nature of motor impairment following TBI is critical, as impaired motor control following concussion has been shown to increase risk for subsequent musculoskeletal injury (18) and falling (37). TBI is also linked to an elevated risk of developing neurodegenerative diseases associated with motor symptoms, including motor neuron disease (38) and Parkinson's disease (PD) (39). For example, multiple studies have established a link between TBI and the later development of PD, with Gardner et al. (40) recently reporting that mild TBI increases risk of PD by 56%, while moderate/severe TBI increases PD risk by 83%. More recently, Russell and colleagues reported in a retrospective cohort study that Scottish former rugby players had a higher incident rate of neurodegenerative diseases, including both PD [HR/OR (95% CI) = 3.04 (1.51–6.10)] and motor neuron disease [HR/OR (95% CI) = 15.17 (2.10–178.96)] compared to a matched comparison group from the general population over a 32-year median follow-up period from study entry (11.4 vs. 5.4%) (41). This is consistent with growing neuroimaging evidence that TBI leads to ongoing neurodegeneration. In the months to years following injury, progressive lesion expansion occurs concomitant with white and gray matter atrophy and loss of white matter integrity (42–45). Importantly, structures affected include those critical for motor function, such as the striatum (46), thalamus (47), and cerebellum (47).

Considering the high prevalence of TBI, a fuller description of neuromotor deficits, stratified by motor domain, in the gross or fine motor will provide insight into the global recovery process and rehabilitation needs of persons with TBI. In addition, given that motor function may play a crucial role in linking TBI to the later emergence of neurodegenerative movement disorders, examining specific motor changes that occur long-term following injury could serve as a novel method for identifying the risk of these diseases. As such, the aim of this systematic review was to review all original research reports that assessed chronic motor outcomes following TBI, stratified by injury severity in both preclinical models and patient populations.

## 2. Methods

### 2.1. Search strategy

The Preferred Reporting Items for Systematic Reviews and Meta-Analysis (PRISMA) guidelines (48) were used. A comprehensive literature search was performed in May 2019, with an updated search undertaken in March 2022, using the electronic databases PubMed, Embase, Scopus, and PsycINFO to identify relevant publications. The search strategy was developed based on an initial scoping search and in consultation with a health and medical sciences librarian. The search terms used were “traumatic brain injury”, “Parkinson's disease”, “motor neuron disease” and “motor performance,” or variations thereof that were combined using “AND” and “OR” search operators. The developed search strategy is depicted in Supplementary Table 1. Further searches were performed in the reference lists from included studies.

### 2.2. Study selection: inclusion and exclusion criteria

**Figure 1:**
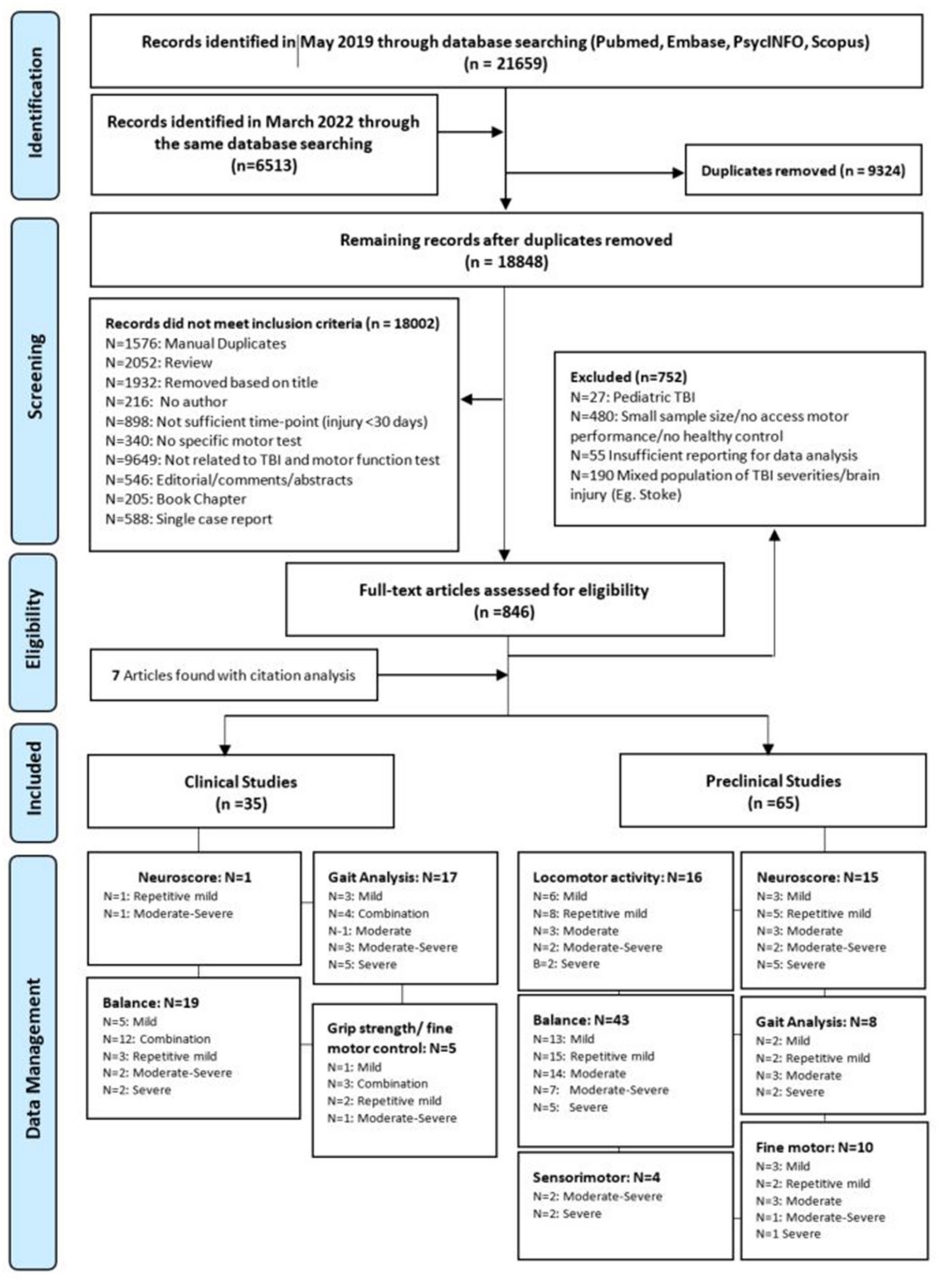
PRIMSA flow diagram outlining the article selection and screening process and subsequent data management. The sum of each domain indicates the total number of publications investigated that motor function. One study could report more than one injury severity or more than one motor functional test.

Following the search, identified articles were imported into EndNote X9.3.3 and duplicates were removed either by the EndNote “delete duplicates” function or deleted manually. Titles and abstracts were then screened, with clinical studies reporting motor outcomes >1 year post-injury and preclinical studies reporting motor outcomes >30 days post-injury retained. For articles that passed this preliminary assessment, the full-text article was retrieved and screened for eligibility against the inclusion and exclusion criteria. The eligibility of articles was assessed by two independent reviewers. Any conflicts were resolved via discussion, and if a consensus could not be reached, a third reviewer was consulted. A flowchart with reasons for the exclusion of studies is displayed in Figure 1.

### 2.3. Inclusion criteria

The following inclusion criteria were utilized:

(i) An original research article published in English.
(ii) Investigated an adult population (preclinical: 8 weeks or older; clinical: 18 years or older) with a prior history of TBI.
(iii) Assessed long-term motor performance (preclinical: >30 days post-injury; clinical: mean time since TBI > 1 year).
(iv) Clear classification of TBI severity [Preclinical: required a comprehensive description of the TBI model and parameters used to induce injury; clinical: Provided Glasgow Coma Scale (GCS), Westmead Post-Traumatic Amnesia scale (PTA), and/or loss of consciousness (LOC) duration].
(v) Compared motor performance with a control group.

The search had no restrictions on the year of publication; however, only English language publications were included. Databases were searched from inception.

### 2.4. Exclusion criteria

Studies were excluded from further consideration as follows:

(i) Reported outcome measurements that were not purely motor (e.g., cognition, visuomotor integration/coordination, social preference, or quality of life).
(ii) Did not specifically state month/time post-TBI, injury severity, or motor outcomes assessed.
(iii) Pilot studies that had a sample size of a single group of less than 5.
(iv) Studies were single case reports/expert options.
(v) Studies were review articles or conference abstracts.
(vi) No specific statistical comparison was reported for injured compared to sham/naïve animals in treatment studies, with treatment effects not the focus of the current review.

### 2.5. Data extraction and synthesis

Data were extracted from the included studies by two independent reviewers. Data extracted included study characteristics (author, year of publication, study design, motor function measurement, injury method in preclinical studies, description of TBI severity for clinical studies); participant/TBI preclinical model characteristics (sample size, age, sex, history of TBI/frequency of injury, time point assessed post-injury, mechanism of injury, and TBI severity); primary methods/functional tests used to measure motor performance, and primary or secondary outcome(s) of motor performance. A copy of the data extraction template is found in Supplementary Table 2.

Due to the large diversity of motor outcome measures used across the study, the measurements were categorized into different motor functions and analyzed separately. The categorization of the motor outcome measures is outlined in Supplementary Table 3. In order to assess the effect of TBI severity on motor performance, outcomes were further stratified by injury severity. The evaluation of TBI severity was classified as described in Supplementary Table 4. Injury severity in preclinical studies was separated into five groups: (i) single mild; (ii) repetitive mild; (iii) moderate; (iv) moderate–severe; and (v) severe. A similar classification system was used for clinical TBI, with minor modifications. As some individuals had experienced more than one injury, the following groups were used: (i) single mild, (ii) the combination of single and repetitive mild (prior TBI history ranged from 1 to more), (iii) repetitive mild (>1 prior TBI), (iv) moderate–severe, and (v) severe.

### 2.6. Assessment of methodological quality

Articles included in the study were assessed for methodological quality by one reviewer (IW), with confirmation provided by a second reviewer (LCP or FC), by using the Systematic Review Center for Laboratory Animal Experimentation (SYRCLE) Risk of Bias tool (preclinical) (49) and the Cochrane Risk of Bias Tool (clinical) (50). Studies were judged as having a low, unclear, or high risk of bias in the following domains: selection bias, performance bias, detection bias, attrition bias, reporting bias, and other biases. The overall risk of bias for each included study was categorized as “strong quality” if the risk of bias was low in 70% or more of the criteria, “low quality” if the risk of bias was high in at least 30% of the criteria, and “moderate quality” if the risk of bias fell between these two parameters. Disagreements were resolved by consensus. Summary graphs were created in Review Manager (RevMan) ([Computer program], Version 5.4.1 Copenhagen: The Nordic Cochrane Center, The Cochrane Collaboration, 2014).

## 3. Results

### 3.1. Search outcomes

The initial search yielded 28,172 articles. From these, 9,324 duplicates were removed with the Endnote function, and another 18,002 were excluded after reviewing the title and abstracts (Figure 1). Full-text analysis was then performed on the remaining 846 articles, of which only 93 met inclusion criteria, with seven additional articles identified from a search of the citation lists of included studies. All articles (*n* = 100) were then separated into preclinical (*n* = 65) and clinical (*n* = 35) subgroups for further analysis. Stratification based on motor outcomes and injury severity was performed as described above. Details of this process are described in the PRISMA diagram (Figure 1).

### 3.2. Study characteristics

**Figure 2:**
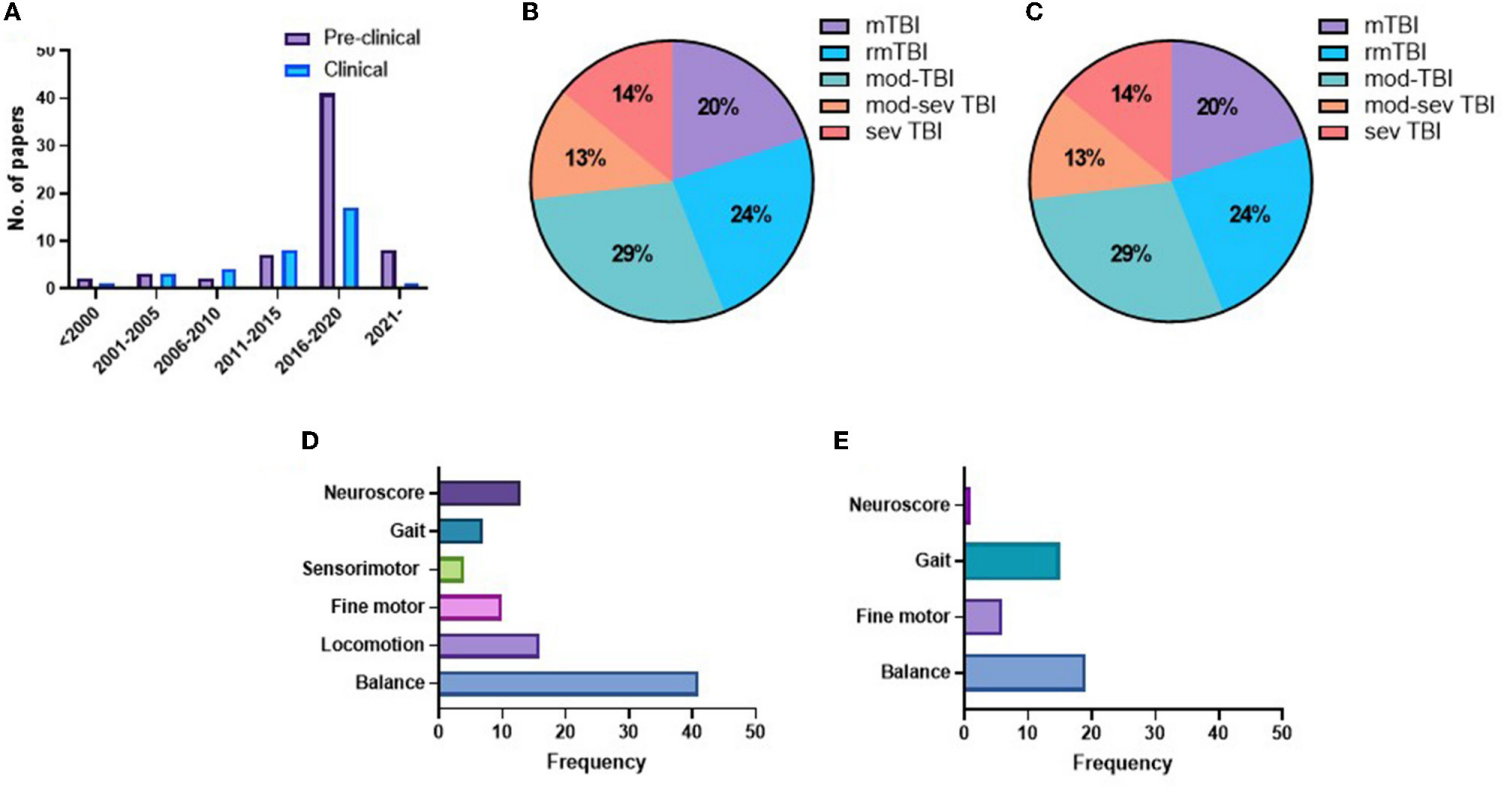
Number of publications per year **(A)**. Pie charts indicating the representation of different injury severities in preclinical **(B)** and clinical studies **(C)**. Frequency charts indicating the number of studies that reported on each motor domain in preclinical **(D)** and clinical studies **(E)**.

The earliest included study was published in 1997, and the rate of publications/year was low until 2016, when the rate of publications increased markedly, particularly for preclinical studies (Figure 2A). In the preclinical studies, there was an even split between the use of mice (50%) and rats (50%). The vast majority of preclinical studies used male animals (82%), with only 11.3% reporting the use of female animals, a further 1.7% using both sexes and four studies (5%) not mentioning the sex of the animals used. In clinical studies, the majority of studies (23/35) had more than 60% male TBI participants (19, 22–26, 40, 51–66), including five studies with only male participants (19, 25, 26, 55, 64), nine studies had a TBI cohort with 70–45% female participants (67–75), and three studies did not state the sex of TBI participants (76–78).

Sample sizes varied across the studies ranging from 6 to 49 animals in a group in preclinical studies. The sample size for the included clinical studies was consistently low, with the majority ranging from 16 to 66 participants, with only a few moderately sized (111–453 individuals)^32 − 395 − 125 − 125 − 125 − 12^ and one large study (4,007 subjects) (55). The source populations of clinical studies varied widely including rehabilitation institutes (22, 23, 59, 67), professional athletes (25, 26), college students (54, 71, 78), college athletes (19, 60, 61, 63, 68, 73, 76), military veterans (40, 52–56, 65, 69), and the community (51, 54, 58, 64, 70, 72, 75, 77).

In preclinical studies, there was a relatively even split across the injury severities, with 20% mTBI, 24% rmTBI, 29% moderate TBI, 13% moderate–severe TBI, and 14% severe TBI (Figure 2B). In comparison, the majority of clinical studies examined a combination of single and repetitive mild TBI (42.5%), with a further 17% reporting single mTBI and 12.5% rmTBI. Moderate–severe TBI was included in only 12.5% of studies and severe TBI in just 15% of studies (Figure 2C). The motor domain most commonly examined in preclinical studies was balanced (43/63 studies), with a much smaller number examining locomotion (16), neuroscore (15), fine motor (10), and gait (8) (Figure 2D). In contrast, in clinical studies, balance (19) and gait (15) were most commonly tested, with fewer studies investigating fine motor control (6) or a neuroscore (1) (Figure 2E).

### 3.3. Risk of bias

**Figure 3:**
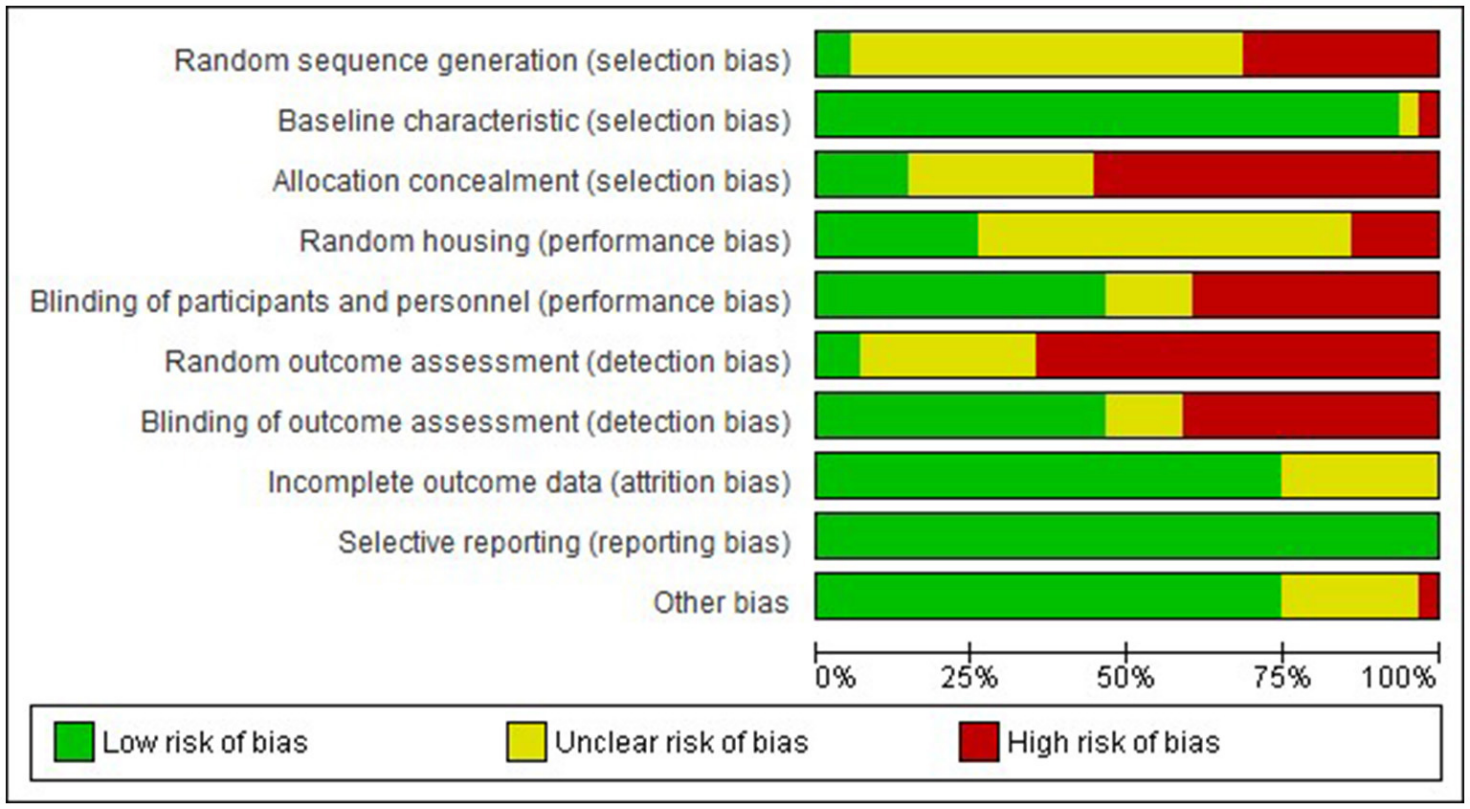
Risk of bias graph-Preclinical. Review authors' judgement about each risk of bias item presented as percentages across all included studies.

**Figure 4:**
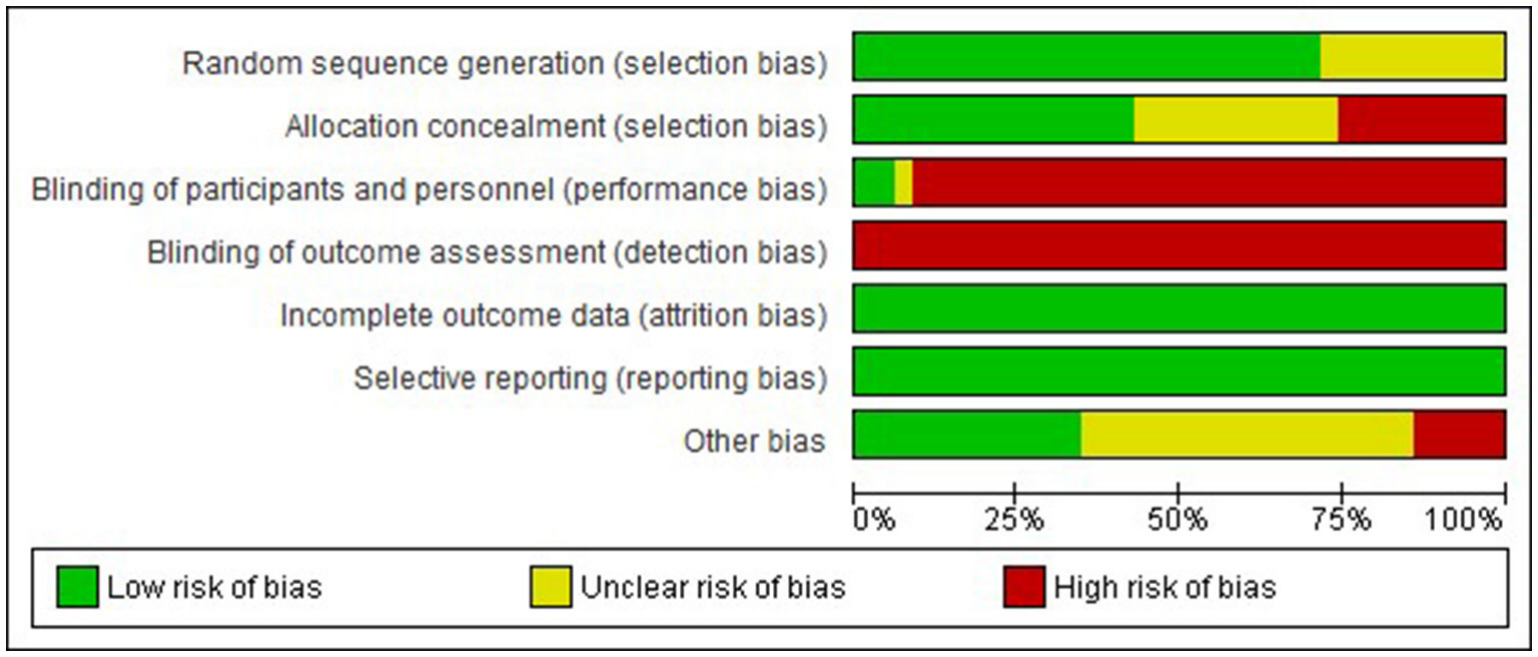
Risk of bias graph-Clinical. Review authors' judgement about each risk of bias item presented as percentages across all included studies.

Examination of risk of bias found that of the preclinical studies, only seven were of strong quality, 24 were of moderate quality, and 31 studies were of low quality (Supplementary Figure 1 for complete evaluation and Figure 3 for summary data). Of the clinical studies, only one was of strong quality, one of moderate quality, and the remaining 32 were of low quality (Figure 4). Key sources of bias for preclinical studies included selection bias and detection bias, whereas for clinical studies, they included selection, performance, and detection bias.

### 3.4. Preclinical motor outcomes

#### 3.4.1. Neuroscore

Overall, a focal injury was required to lead to persistent decreases in neuroscore, with minimal changes seen with diffuse injury. It should be noted the neurological severity scoring system varied widely among the 15 included studies, with the standard neurological severity score (79–85), modified neurological severity score (86–88), and revised neurological severity score (89) all represented. Even articles that used the same names for their scoring systems incorporated different tasks within their behavioral battery (Supplementary Table 4). The most commonly included items were those from the standard neurological severity score, including forelimb and hindlimb flexion on tail suspension (10 of 15 studies) (79, 81–85, 87, 89–91), forelimb/hindlimb placement on a flat surface (5/15) (79, 83, 84, 87, 90), and resistance to lateral pulsion (6/15) (79, 82, 84, 85, 90, 91). Other tests that were included were simple reflexes (limb, tail, corneal, and startle), with different combinations used in seven studies (30, 80, 81, 87–89, 92). Measures of hemiparesis, either via direct assessment of hemiplegia (30, 80, 86) or circling behavior (80, 86, 87, 91), were included in six studies. Balance was examined as ability to stay on a round beam (80, 86, 87), a flat surface (80, 86), or an inclined plane (79, 82, 83), as well as the ability to walk across beams of different widths (1, 2, and 3 cm) (80, 86, 88, 89, 92), with a measure of balance incorporated into nine of the 15 studies. General activity was assessed either directly (90) or via ability to exit a circle (86) or seeking behavior (86, 92) in three studies. Direct assessment of gait as performance on the treadmill was included in one study (30). Given the different combinations of tests which reflect different types of motor behaviour were included in the neurological severity score across the studies, direct comparisons between studies is difficult. Nonetheless, in studies utilizing focal or mixed moderate (30, 81, 87), moderate–severe (90, 91), and severe TBI models (82–85, 92), a consistent impairment was found in neuroscore, regardless of the scoring system implemented, out to 8 months post-injury, with no further deficits noted in the one study that included a 12-month time point (Table 1) (82). In general, no chronic deficits in neuroscore were noted in preclinical models of smTBI (79, 80) or rmTBI (79, 80, 86, 89), with 7 months as the latest time point examined. Only one study utilizing a projectile concussive impact where a steel ball is projected at the rat head which is protected by a steel helmet found chronic neuroscore deficits at 3 months post-injury in both single and repeated (4 x mTBI 1 h apart) animals (88).

**Table 1:** Preclinical neurological severity score evaluation.

**References**	**Years**	**Method**	**Severity**	**Type injury**	**Sample**	**Pre**	**<72h**	**1–3wks**	**1–2mo**	**3–5mo**	**6–11mo**	**12–24mo**
Laurer et al. (79)	2001	CCI-CS	Mild	Diffuse	T:16–24; C:14–23		^**+**^	-	-			
Fehily et al. (80)	2019	WD	Mild	Diffuse	T:15; C:15					-		
Mountney et al. (88)	2017	PCS	Mild	Diffuse	T/C: 8–10					^+^		
Mountney et al. (88)	2017	PCS	Rep-Mild	Diffuse 4 × TBI/1hr	T/C: 8–10					^+++^		
Feng et al. (89)	2021	CHIMERA	Rep-Mild	Diffuse 3xTBI/day × 2 48 hrs	T:9–13; C:12–14		-	-	-			
Laurer et al. (79)	2001	CCI-CS	Rep-Mild	Diffuse 2xTBI 24 hrs	T:16–24; C:14–23		^+++^	^+++^	-			
Fehily et al. (80)	2019	WD	Rep-Mild	Diffuse 2xTBI 24 hr	T:15; C:15					-		
			Rep-Mild	Diffuse 3xTBI 24 hr	T:15; C:15					-		
Huynh et al. (86)	2020	CCI-CS	Rep-Mild	Left sided 5xTBI, 48 hr	T:15; C:15					-	-	
Zhang et al. (87)	2021	CCI	Moderate	Focal	T:10; C:10		^++^	^++^	^++^			
Daglas et al. (30)	2019	CCI	Moderate	Focal	T:12; C:12		^++++^	^++++^	^++++^	^++++^	^++++^	
Sell et al. (81)	2017	LFP	Moderate	Mixed	T:11–14; C:12–14		^+++^	/	/	^+++^	-	-
Shear et al. (91)	2010	PBBI	Mod-Sev	Focal	T/C=10		^+^	^+^	^+^			
Wang et al. (90)	2019	Punch	Mod-Sev	Focal	T:8; C:8		^++^	^++^	^++^			
Segovia et al. (85)	2020	LFP	Severe	Mixed	T:7; C:7	-	^+++^		^+++^			
Nissinen et al. (84)	2017	LFP	Severe	Mixed	T:35; C:16	-	^+++^	^+++^	^+++^			
Zhau	2021	CCI	Severe	Focal	T:10; C:10		^+++^	^+++^	^+++^			
Zhang et al. (83)	2005	LFP	Severe	Mixed	T:14; C:24			^++^	^++^	^++^		
Pierce et al. (82)	1998	LFP	Severe	Mixed	T:12–16; C:11–15		^+^	^+^	^+^		^+^	-

^+^*p* < 0.05; ^++^*p* < 0.01; ^+++^*p* < 0.001; ^++++^*p* < 0.0001; -, not significant; Gray, not evaluated. CCI-CS, controlled cortical impact-closed skull; WD, weight drop; CHIMERA, Closed-Head Impact Model of Engineered Rotational Acceleration; LFP, lateral fluid percussion; CCI, controlled cortical impact; PBBI, penetrating ballistic like impact.

#### 3.4.2. Gait analysis

Gait was analyzed post-TBI using automated systems such as the CatWalk, requiring animals to actively walk across a platform (28, 29, 31, 88, 93, 94), or the DigiGait apparatus, which utilizes a treadmill at a steady speed (27, 30). In general, the studies were selective in reporting gait parameters, not providing a comprehensive overview of the different measures in the methods and often only reporting selective results, with some exceptions (88) (Table 2). No single impact study reported chronic changes in speed or cadence following TBI, with this either directly reported (29, 31, 94), or not included within the results (27, 28, 30, 93), with an acute decrease in speed only noted at 24 h following severe focal injury (31). Indeed persistent reductions in cadence were only noted when four impacts were delivered 1 h apart, with this deficit persisting to 3 months post-injury (88).

**Table 2:** Preclinical gait evaluation.

**References**	**Years**	**Method**	**Severity**	**Type injury**	**Apparatus**	**Parameters examined**	**Sample**	**Pre**	** < 72h**	**1–3wk**	**1–2mo**	**3–5mo**	**6–11mo**	**12–24mo**
Namdar et al. (93)	2020	WD	Mild	Diffuse	CatWalk	Front base of support	T:15; C:13			^+^	^+++^			
						Standing on diagonal two				^+^	-			
						Standing on three				^++^	^+^			
						Hind base of support				-	-			
Mountney et al. (88)	2017	PCI	Mild	Diffuse	CatWalk	Stand (sec)	T/C: 8–10		^+^	-	-			
						Stand index			^+^	-	-			
						Swing (sec)			-	-	-			
						Step cycle (sec)			-	-	-			
						Single stance			-	-	-			
						Stride length			-	-	^+^			
						Front base of support			^++^	^+^	^+++^			
						Hind base of support			-	-	^+++^			
						Three limb support			-	-	-			
						Cadence			-	-	-			
Bolton	2016	CCI-CS	Rep-Mild	5 × TBI 24 hrs	Digigait, treadmill 15 cm/s	Gait symmetry	T/C: 10		-		-			
						Hindlimb shared stance			-		-			
						Paw contact area			-		-			
Bolton et al. (27)	2016	CCI-CS	Rep-Mild	5 × TBI 48 hrs	Digigait, treadmill 15 cm/s	Gait symmetry			-		-			
						Hindlimb shared stance			-		-			
						Paw contact area								
Mountney et al. (88)	2017	PCI	Mild	Diffuse 4 × TBI/1hr	CatWalk	Stand (sec)	T/C: 8–10		^+++^	^+^	-			
						Stand index			^++^	^++^	^++^			
						Swing (sec)			^+++^	^+^	^+^			
						Step cycle (sec)			^+++^	^+++^	^+^			
						Single stance			^+++^	^+^	^+^			
						Stride length			-	^+^	-			
						Front base of support			^++^	^+^	^+++^			
						Hind base of support			-	-	^+++^			
						Three limb support			^++^	^+^	-			
						Cadence			^+++^	^+++^	^++^			
Henry et al. (28)	2020	CCI	Moderate	Focal	CatWalk	Paw contact area	T:12; C:12	-	-	-	-			
						Stance, swing								
						Speed								
						Interlimb coordination								
						Base of support								
						%Support time								
Ritzel et al. (29)	2020	CCI	Moderate	Focal	CatWalk	Step Sequence	T/C: 16–23						^+^	
						Stride length (RH)							^+^	
						Swing speed (RF,RH)							^+^	
						Print position							^+^	
						Average speed, number of steps							-	
Daglas et al. (30)	2019	CCI	Moderate	Focal	DigiGait 15 cm/s	Swing duration (RH)	T:12; C:12	-		^+^	^+^	^+^	^++^	
						Propulsion duration (RF)		-		-	^++^	^+^	^+++^	
Cline et al. (31)	2017	CCI	Severe	Focal	CatWalk	Cadence	T:15; C:14		^++^		-			
						Average Speed			^++^		-			
						Swing duration (RH)			^++^		^+^			
						Average Swing speed (LF, RF, RH)			^+^		-			
Schönfeld et al. (94)	2017	CCI	Severe	Focal	CatWalk	Stride length Base of support Three limb support; Speed Cadence	T:10; C:7	-		-	-			

^+^*p* < 0.05; ^++^*p* < 0.01; ^+++^*p* < 0.001; -, not significant; Gray, not evaluated. T, TBI; C, control; RH, right hindlimb; RF, right forelimb; LF, left forelimb; CCI-CS, controlled cortical impact-closed skull; WD, weight drop; PCI, projectile concussive impact.

However, following even single mTBI, subtle gait alterations were noted with free ambulation of the CatWalk including a decreased front base of support seen acutely and persisting up to 3 months post-injury (1, 2). Namdar et al. (93), with a decrease in hind base of support also developing at the 3-month time point (88). Repeated injury led to more prominent gait abnormalities, with the 4 × 1 h apart injury model leading to persistent deficits to 3 months post-injury in single-stance time, stride length, stand time, and front base of support, with the deficit in hind base support again only developing at 3 months post-injury (88). In contrast, on a treadmill, repeat injury (5× mTBI 24 or 48 h apart) led to no deficits in gait either acutely or at 1 month, although different gait parameters were reported across studies, including gait symmetry, paw contact area, and hindlimb shared stance (27).

Mixed results were also found for gait following more severe injury. Following focal moderate CCI injury, no deficits were detected from 24 h to 1 month post-injury on the CatWalk, with reporting of measures like paw contract area, stance, swing speed, base of support, and interlimb coordination (28). In contrast, Ritzel et al. only examined gait at 26 weeks post-moderate CCI injury and reported a number of impairments, including reduced stride length of the contralateral right hindlimb and reduced swing speed in the right hind and forelimb, but no overall change in average speed (29). Similarly, Daglas et al. utilizing the DigiGait treadmill apparatus found significant reductions in contralateral swing duration of the right hindlimb with compensatory right forelimb propulsion duration from 1–32 weeks post-moderate CCI injury (30). Surprisingly, minimal chronic deficits were reported following severe CCI injury, with Cline et al. only detecting deficits at 24 h, but not 1 month, post-injury, with the exception of swing duration of the contralateral hindlimb (31). Similarly, Schönfeld et al. also found no alterations in stride length, base of support, or three limb support at 1 month following injury (94).

#### 3.4.3. Sensorimotor control

The sensorimotor function was primarily evaluated with the adhesive removal test (1–4), with severe injury required to produce chronic deficits. The number of trials evaluated varied between studies ranging from 1 to 6, as did the presentation of results, which included % sham (95), latency to remove the adhesive (83, 96), and difference in performance between preferred and non-preferred paw (94) (Table 3). Severe injury, either focal (94) or mixed (83), led to persistent deficits in adhesive removal out to 16 weeks post-injury, the latest time point assessed. In contrast, following moderate–severe injury, neither focal (95) nor diffuse (96) injury led to chronic deficits in adhesive removal, with deficits noted up to 6 days following diffuse injury and 3 weeks following focal injury, with no further impairment noted to 41 days post-injury, the latest time point assessed. Interestingly, the whisker-evoked forepaw placement task did detect chronic deficits in the diffuse injury moderate–severe model with impaired forepaw placement out to 41 days post-injury (96), indicating potential task-dependent effects. To date, no studies have evaluated sensorimotor function chronically following either a single mTBI or repeated mTBI.

**Table 3:** Preclinical sensorimotor evaluation.

**References**	**Years**	**Method**	**Severity**	**Type Injury**	**Test**	**Parameters examined**	**Sample**	**Pre**	** < 72h**	**1–3wks**	**1–2mo**	**3–5 mo**	**6–11 mo**	**12–24mo**
Hoffman et al. (95)	2003	CCI	Mod-Sev	Focal	Adhesive Test	3 × 2 min trials %sham	T:8; C:8			^+^	-			
Alwis et al. (96)	2012	WD	Mod-Sev	Diffuse	Adhesive Test	1 trial Latency	T:19; C: 12	-	^+^	-	-			
					Whisker-evoked forepaw placement	10 trials No. correct	T:4; C:4		^+^	^+^	^+^			
Schönfeld et al. (94)	2017	CCI	Severe	Focal	Adhesive Test	3 trials Paw difference	T:10; C: 6	-		^+^	^+^			
Zhang et al. (83)	2005	LFP	Severe	Mixed	Adhesive Test	6 trials Latency	T:14; C:13			^++^	^++^	^++^		

^+^*p* < 0.05; ^++^*p* < 0.01; -, not significant; Gray, not evaluated. T, TBI; C, control/; CCI, control cortical impact; WD, weight drop injury; LFP, lateral fluid percussion injury.

#### 3.4.4. Grip strength and fine motor

Grip strength and fine motor ability were assessed via the grip strength meter (30, 93, 97–100), isometric pull task (33, 101), pellet reaching tasking (34), and Montoya staircase test (94) (Table 4). The grip strength meter requires minimal pre-training, whereas the other tasks involve more extensive training with food restriction, with the test relying on the animal's desire to obtain sugar pellets as a reward for completing the task successfully. Overall, with single diffuse injury, moderate (32, 33, 101), but not mild, injury (93, 97, 98) led to chronic grip strength deficits. However, the protocol for assessing grip strength varied between studies, with the number of trials examined ranging from 1 to 10. Following diffuse single mTBI, no deficits were detected on grip strength up to 12 weeks post-injury (93, 97, 98), with only one study finding an acute deficit at 1 week post-injury (98). In contrast, diffuse moderate TBI did produce deficits in grip strength at 28 days post-injury, the latest time point assessed (32, 33, 101). For repeated mild injury, bilateral mTBI weekly for 5 weeks led to impaired grip strength at 40–45 weeks post-injury (100), whereas 3 impacts 24 h apart led to no deficits at 30 days post-injury, (99) with these the only time points assessed in these studies. Notably, with the negative result, the average of three trials relative to body weight was recorded (99), whereas the positive result was only one trial reported as maximum force achieved, introducing a potential confound (100). To date, no study has assessed grip strength alterations chronically following a severe TBI. Another test of strength, the isometric pull task, was used in two studies of focal moderate TBI, in which the lesion was specifically located over the motor cortex (33, 101). In this task, rats were trained prior to injury to reach a force threshold of at least 120 g within 2 s on a pull lever in order to receive a food reward. Following injury, rats had a decrease in maximal force produced, a decrease in the % of successful trials and decrease in the speed of force generation from weeks 1–6 post-injury (33, 101). However, in successful trials, the time taken to reach the 120 g threshold was only significantly increased in Weeks 1–2, returning to sham level from Week 3. This mirrored results for total trials, which were significantly decreased from Weeks 1–2 before returning to sham level, potentially indicating reduced motivation as well (33). However, given the focal nature of the injury in these studies, it is difficult to know whether these findings would transfer to a diffuse injury model.

**Table 4:** Preclinical grip strength and fine motor evaluation.

**References**	**Years**	**Method**	**Severity**	**Type Injury**	**Test**	**Parameters examined**	**Sample**	**Pre**	** < 72h**	**1–3 wks**	**1–2mo**	**3–5mo**	**6–11mo**	**12–24mo**
Evans et al. (98)	2014	CCI-CS	Mild	Diffuse	Grip strength meter	Average 10 trials	T9; C8		-	^+^	-	-		
Namdar et al. (93)	2020	WD	Mild	Diffuse	Grip strength meter	5 trials, average best 3	T:10; C:8			-	-			
Evans et al. (97)	2015	CCI-CS	Mild	Diffuse	Grip strength meter	Average 10 trials	T:12; C:11		-	-	-			
Tabet et al. (99)	2022	CCI-CS	Rep-Mild	3 × TBI 24 hrs	Grip strength meter	Average 3 trials relative to weight	T:11; C:11				-			
Dhillon et al. (100)	2020	CCI-CS	Rep-Mild	2xTBI (L^+^R) 5 × weekly	Grip strength meter	1 trial	T:10; C:8						^+^	
Rana et al. (32)	2020	WD	Moderate	Diffuse	Grip strength meter	1 trial	T:7; C:5			^+^	^+^			
Pruitt et al. (33)	2014	CCI	Moderate	Focal (motor cortex)	Isometric pull task	Maximal Force	T:15; C:11	-		^+^	^+^			
						% Successful Trials		-		^+^	^+^			
						Time to 120 g threshold		-		^+^	-			
						Speed force generation		-		^+^	^+^			
						Total Trials		-		^+^	-			
Pruitt et al. (97)	2017	CCI	Moderate	Focal (motor cortex)	Isometric pull task	Maximal Force	T:6; C: 6	-		^+^	^+^			
						% Successful Trials				^+^	^+^			
Adkins et la. (34)	2015	CCI	Mod-Sev	Focal	Pellet reaching Test	% successful	T:41; C:31	-	^+++^	^+++^	^+++^			
Schönfeld et al. (94)	2017	CCI	Severe	Focal	Montoya staircase test	Pellets eaten	T:8; C:7	-		^+++^	^++^			

^+^*p* < 0.05; ^++^*p* < 0.01; ^+++^*p* < 0.001; -, not significant; Gray, not evaluated. T, TBI; C, control/Sham; CCI, control cortical impact; CCI-CS, control cortical impact-closed skull; WD, weight drop injury.

Fine motor skills were assessed by Adkins et al. (34) and Schönfeld et al. (94) using variations of a pellet reaching task following moderate–severe and severe focal injury, respectively, with significant deficits found out to 6 weeks post-injury. In the Adkins et al. study, the pellets were located on a flat surface in front of the animals (34), while Schönfeld et al. (94) used a staircase with pellets placed on increasing higher steps to enhance difficulty. In the pellet reaching task, injured animals had a decrease in the % successful reaching attempts to 42 days post-injury (34). In the Montoya staircase task, injured rats obtained significantly fewer pellets across all steps, made less reaching attempts, and misplaced more pellets on the upper steps (94).

#### 3.4.5. Locomotor activity

The open-field test, the most commonly used task to measure general locomotor activity levels by examining the total distance traveled over a test period (5–60 min), was used across all included studies (Table 5). Following diffuse mTBI, either single or repeated, locomotor results varied depending on the species (rat vs. mice), strain, protocol, and apparatus employed (Table 5). The utilization of a smaller apparatus (19 × 11 cm) following weight drop TBI in Swiss mice over a 60-min period found persistent hyperactivity from 48 h to 12 weeks following injury (102). In contrast, closed skull CCI injury in C57BL/6J mice led to no changes in locomotion over 30 min in a larger 49 × 36 cm arena either acutely or chronically up to 90 days post-injury (103). Indeed, repeated impacts over a short interval (Morriss et al.: 5 × 24 h; Tucker et al.: 3 × 24h) were required to replicate this hyperactivity in mice in a larger arena (40 × 40 cm), with this behavior developing at 3 months post-injury (104, 105) and persisting to 12 months post-injury (105), the latest time point examined. With 2 CCI-CS impacts over 3 days, changes in locomotion were no longer observed in mice in a similar size arena over 30 min on day 1, day 7, or 12 weeks post-injury (103).

**Table 5:** Preclinical locomotor activity evaluation.

**References**	**Year**	**Method**	**Severity**	**Type injury**	**Test**	**Time**	**Size**	**Sample**	**Pre**	** < 72 hr**	**1–3 wks**	**1–2 mo**	**3–5 mo**	**6–11 mo**	**12–24 mo**
Namdar et al. (93)	2020	WD	Mild	Diffuse	Open Field	10 mins	60 × 60 cm	T:15; C:13			-	-			
Homsi et al. (102)	2010	WD	Mild	Diffuse	Open Field	60 mins	19 × 11 cm	T/C: 10–12		^+++^	^++^	^++^	^+^		
Bajwa et al. (103)	2016	CCI-CS	Mild	Diffuse	Open Field	30 mins	49 × 36 cm	T:10; C:10		-	-		-		
McAteer et la. (106)	2016	WD	Mild	Diffuse	Open Field	5 mins	1 × 1 m	T:9; C: 9				^+^	-		
Arulsamy et al. (108)	2019	WD	Mild	Diffuse	Open Field	5 mins	1 × 1 m	T:14; T:14							-
Arun et al. (114)	2020	Blast	Mild	Blast	Open Field	60 mins	40 × 40 cm	T/C: 10–31		-	-	-	-	^+^	-
Feng et al. (89)	2021	Chimera	Rep-Mild	Diffuse 2 × 3 d	Open Field	Unknown	40 × 40 cm	T^+^C:81		-	-	-			
Bajwa et al. (103)	2016	CCI-CS	Rep-Mild	Diffuse 2 × 3 d	Open Field	30 mins	49 × 36 cm	T:10; C:10		-	-		-		
Corrigan et al. (107)	2017	WD	Rep-Mild	Diffuse 3 × 5 d	Open Field	5 mins	1 × 1 m	T/C: 8–10					^+^		
McAteer et al. (106)	2016	WD	Rep-Mild	Diffuse 3 × 5 d	Open Field	5 mins	1 × 1 m	T:7; C: 9				^+^	^+^		
Morriss et al. (104)	2021	WD	Rep-Mild	Diffuse 5x 24 hrs	Open Field	Unknown	Unknown	T:11; C:10				-	^+^	^+^	
Arulsamy et al. (108)	2019	WD	Rep-Mild	Diffuse 3 × 5 d	Open Field	5 mins	1 × 1 m	T:14; C:14							-
Arun et al. (114)	2020	Blast	Rep-Mild	Blast 2 × 2 mins	Open Field	60 mins	40 × 40 cm	T/C: 10–31		^++^	-	-	^+^	^++^	^++^
Tucker et al. (105)	2019	CCI-CS	Rep-Mild	Diffuse 3x 24 hrs	Open Field	20 mins	40 × 40 cm	T/C: 17–21				-	^+++^	^+++^	^+++^
Bajwa et al. (103)	2016	CCI	Moderate	Focal	Open Field	30 mins	49 × 36 cm	T:10; C:10		-	^+++^		-		
Leconte et al. (109)	2020	CCI	Moderate	Focal	Open Field	9 mins	1 m × 1 m	T:15; C:13					^+^		
Rowe et al. (111)	2016	LFP	Moderate	Mixed	Open Field	5 mins	70 × 70 cm	T/C:11–12: 2M				-	-	-	
								T/C:11–12: 4M				-	-		
								T/C:11–12: 6M				-			
Arulsamy et al. (113)	2018	WD	Mod-Sev	Diffuse	Open Field	5 mins	1 x1 m	T:14; C:13				-	^+^		
Arulsamy et al. (108)	2019	WD	Mod-Sev	Diffuse	Open Field	5 mins	1 x1 m	T:12; C: 14							-
Islam et al. (110)	2021	CCI	Severe	Focal	Open Field	5 mins	54.5 × 54.5 cm	T,C: 9–13					^+^		
Komoltsev et al. (112)	2021	LFP	Severe	Mixed	Open Field	5 mins	1 × 1 m	T:13; C:7						-	

^+^*p* < 0.05; ^++^*p* < 0.01; ^+++^*p* < 0.001; -, not significant; Gray, not evaluated. T, TBI; C, control; CCI, control cortical impact; WD, weight drop injury; LFP, lateral fluid percussion injury.

Varied results have been reported in rat studies. Mild weight drop TBI in Sprague-Dawley rats found no difference in locomotion in a 60 × 60 cm arena over 10 min at either 1 or 4 weeks post-injury (93), although a decrease in locomotion has been reported at 6 weeks, resolving by 12 weeks post-injury in a larger 1 × 1 m enclosure over 5 min (106). In contrast, with repeated injury (3 × 5 days), both a decrease in locomotor activity at 6 and 12 weeks (107) and an increase in locomotor activity at 12 weeks (107) have been reported utilizing the same testing parameters. Notably, the decrease was recorded with manual counting of squares crossed (106), whereas the increase was detected using automated software of distance traveled (107). Nonetheless, no differences in locomotion were noted at 12 months post-injury in the same injury model (108), nor in a repeated CHIMERA model (2 × 3 days) in a smaller open field (40 cm × 40 cm) from day 1 to 12 weeks post-injury (89).

Increasing injury severity had little effect on chronic locomotor activity. Moderate focal CCI injury in C57/BL6 mice found a transient decrease in distance traveled at 7 days, but this resolved by 12 weeks (103). Leconte et al. similarly showed no difference compared to naïve animals at 5 months following CCI injury in rats (109). Even with more severe injury, locomotor performance was unchanged at 10 weeks following injury in young mice, with a significant difference only seen in mice injured at 18 months of age (110). Similarly, mixed focal/diffuse injury via LFP had no effect on locomotor activity, as measured via distance traveled over 5 min out to 6 months post-injury (111, 112). The pattern of deficits differed slightly with moderate–severe diffuse injury, with no differences noted at 4 weeks, a decrease in distance traveled at 12 weeks (113), with recovery by 12 months, the latest time point examined (108). A similar pattern was seen following a single blast injury (114). No changes were seen from day 1 to 3 months post-injury, but this was followed by a subsequent significant decrease in total distance traveled over 60 min in a 40 × 40 cm arena at 6 and 9 months, with recovery by 12 months post-injury (114). Indeed, two 19 PSI injuries delivered within 2 min were required to lead to a persistent decrease in locomotor activity at 12 months, with deficits seen within the first 3 days post-injury, resolving at 4 weeks post-injury, prior to re-emerging at 3 months, and then persisting to the 12 month time point (114).

#### 3.4.6. Balance and coordination

Balance and coordination were the most common motor domains evaluated in preclinical studies via tasks encompassing the balance beam (28, 81, 83, 84, 90, 96, 100, 103, 111, 115–122), ladder, (80, 93, 99, 123) rotating pole (79, 83, 114), grid walk, (31, 103), string suspension (124), pole climbing (99, 109), and rotarod tasks (28, 35, 36, 83, 88–90, 93, 96–98, 100, 103–106, 113–115, 119, 124–132) (Table 6). The tests conducted varied between studies including variation in the size of the beam and speed of the rotarod and rotating pole. Furthermore, the parameters examined varied between studies. For example, beam performance was analyzed via time to traverse beam (81, 111, 117, 119, 121), number of foot faults (28, 31, 79, 103, 111, 118, 119, 122), or a ranking scale for performance (81, 83, 84, 90, 96, 124). For the rotarod, performance was evaluated on one trial (93, 96, 97, 106, 108) or an average across up to eight trials (35, 36, 89, 93, 105, 124–126, 131) which may influence results.

**Table 6:** Preclinical balance and coordination evaluation.

**References**	**Years**	**Method**	**Severity**	**Injury**	**Test**	**Parameters**	**Sample**	**Pre**	** < 72h**	**1–3wk**	**1–2mo**	**3–5mo**	**6–11 mo**	**7–11mo**	**12–24mo**
Evans et al. (97)	2015	CCI-CS	Mild	Diffuse	Rotarod (4–40 prm)	Latency to fall	T:12; C: 11		^+^	^+^	-				
Namdar et al. (93)	2020	WD	Mild	Diffuse	Rotarod (4–40 rpm)	Latency to fall	T:15; C:13			-	-				
					Eramus Ladder	Correct steps				-	^+^				
						Missteps				-	^+^				
						Time				-	-				
Lai et al. (117)	2019	WD	Mild	Diffuse	0.5 cm Beam	Traverse time	T:7; C:7	-		^+++^	^+++^				
Laurer et al. (79)	2001	CCI-CS	Mild	Diffuse	3 cm Rotating pole 1,3,5 rpm	Foot-faults	T:16–24; C:14–23		-	-	-				
Bajwa et al. (103)	2016	CCI-CS	Mild	Diffuse	0.65 cm Beam 2.5 cm Grid Walk Rotarod	Traverse Time Foot faults Latency to fall	T:10; C:10		-	-		-			
Evans et al. (98)	2014	CCI-CS	Mild	Diffuse	Rotarod (4–40 prm)	Latency to fall	T:9; C: 8			^+^	^+^	-			
Fehily et al. (80)	2019	WD	Mild	Diffuse	Ladder walk	% Stepping errors	T:15; C:15					-			
McAteer et la. (106)	2016	WD	Mild	Diffuse	Rotarod (3–30 rpm)	Latency to fall	T:9; C:9		-	-	-	-			
Hou et al. (131)	2017	WD	Mild	Diffuse	Rotarod (3–30 rpm)	Average 3 trials	T:8; C: 8	-		-	^+^	^++^			
Mouzon et al. (35)	2014	CCI-CS	Mild	Diffuse	Rotarod (5–50 rpm)	Average 3 trials	T:12; C:12						-		
Mouzon et al. (36)	2018	CCI-CS	Mild	Diffuse	Rotarod (5–50 rpm)	Average 3 trials	T:7; C:8								-
Xu et al. (121)	2019	CCI	Mild	Diffuse	2 cm Beam	Traverse Time	T:10; C10		-	-	-				
Mountney et al. (88)	2017	PCS	Mild	Diffuse	Rotarod (0.1 rpm/sec) Three sets × 5 with 2 min intertrial interval	Latency to fall	T/C: 8–10					-			
Mountney et al. (88)	2017	PCS	Rep-Mild	Diffuse 4xTBI 1 hr apart	Rotarod (0.1 rpm/sec) Three sets × 5 with 2 min intertrial interval	Latency to fall	T/C: 8–10					^+^			
Albayram et al. (124)	2017	WD	Rep-Mild	Diffuse 7 in 9D	0.8 cm beam	Score	T/C: 9–10						^++^		
					String Suspension (3 trials)	Score							^++^		
					Rotarod (4–40 opm) 5 mins; 4x day for 2 days	Latency to fall Average 8 trials							^+^		
Feng et al. (89)	2021	Chimera	Rep-Mild	Diffuse 3xTBI/day × 2 48 hrs apart	Rotarod (5–40 rpm)	Latency to fall- average 3 trials	T:9; C:9				-				
Tabet et al. (99)	2022	CCI-CS	Rep-Mild	Diffuse 3x TBI 24hr	Ladder rung	% Foot faults to baseline	T:10; C:10				^+^				
					Pole climbing	Time (3 trials)	T:10; C:10				-				
Laurer et al. (79)	2001	CCI-CS	Rep-Mild	Diffuse 2xTBI 24 hrs	3 cm Rotating pole	(1, 3, 5 rpm)	T:49; C:36		^++^	-	^++^				
Bajwa et al. (103)	2016	CCI-CS	Rep-Mild	Diffuse 2x 3d	0.65 cm Beam 2.5 cm Grid Walk Rotarod (2x 5 rpm, 2x 3 rpm/5s, 2x 3 rpm/3s	All measures Foot faults Average of trials	T:10; C:10		-	-		-			
Mannix et al. (125)	2014	WD	Rep-Mild	7inj/9D	Rotarod (0.1 rpm/sec)	Average 4 trials	T:32; C:21		^+^			^+^			
Mannix et al. (126)	2017	WD	Rep-Mild	7inj/9D	Rotarod (0.1 rpm/sec)	Average 4 trials	T:12; C:11		^+^			-			
McAteer et la. (106)	2016	WD	Rep-Mild	Diffuse 3 × 5d	Rotarod (3–30 rpm)	Latency to fall	T:7; C:9		-	-	-	-			
Fehily et al. (80)	2019	WD	Rep-Mild	Diffuse 2xTBI 24 hr	Ladder walk	% Stepping errors	T:15; C:15					-			
				Diffuse 3xTBI 24 hr	Ladder walk	% Stepping errors						-			
Mouzon et al. (35)	2014	CCI-CS	Rep-Mild	Diffuse 5xTBI 24 hr	Rotarod (5–50 rpm)	Latency to fall- average 3 trials	T:12; C:12						-		
Morriss et al. (104)	2021	WD	Rep-Mild	Diffuse 5xTBI 24 hr	Rotarod	Latency to fall 4 trials	T:11; C:10							^++^	
Dhillon et al. (100)	2020	CCI-CS	Rep-Mild	Bilateral 5x TBI/5 weeks	Rotarod (3–30 rpm)	Fall- 3 trials	T:10; C:8	-	-	^+^	^+^	^+^	^+^	^+^	^+^
					2.5 cm beam	Hindlimb rating								^+^	
Tucker et al. (105)	2019	CCI-CS	Rep-Mild	Diffuse 3xTBI 24hr	Rotarod (4–60 rpm)	Latency to fall- average 3 trials	T:19–21; C17–19		^+++^		-	^+^	^+^		-
Mouzon et al. (36)	2018	CCI-CS	Rep-Mild	Diffuse 5xTBI 24hr	Rotarod (5–50 rpm)	Latency to fall- average 3 trials	T:7; C:7								-
Hou et al. (131)	2017	WD	Moderate	Diffuse	Rotarod (3–30 rpm)	Average 3 trials	T:8; C: 8	-		-	^+^	^+++^			
Toshkezi et al. (128)	2018	CCI	Moderate	Focal	Rotarod (2–20 rpm)	Latency to fall	T:9; C: 5				^+++^				
Barrett et al. (122)	2020	CCI	Moderate	Focal	0.5 cm Beam	Foot Faults	T/C: 8–13	-	^+++^	^+++^	^+++^				
Henry et al. (28)	2020	CCI	Moderate	Focal	0.5 cm Beam	Foot Faults	T:11; C:12	-	^+++^	^+++^	^+++^				
					Rotarod (1–30 rpm)	%Baseline		-	^+++^	-	^+++^				
Xie et al. (120)	2019	CCI	Moderate	Focal	0.6 cm Beam	Foot Faults	T:10; C:10	-	^+++^	^+++^	^+++^				
Xu et al. (121)	2019	CCI	Moderate	Focal	2 cm Beam	Traverse Time	T/C: 38		-	^+^	^+^				
Chen et al. (123)	2016	NY	Moderate	Focal	Ladder test	Errors	T:20; C:10	-	^++^	-	-				
Bajwa et al. (103)	2016	CCI	Moderate	Focal	0.65 cm Beam	Time Active	T:10; C:10		^+^	-		-			
						Falls			^++^	-		-			
					2.5 cm Grid walk	Foot faults			^+++^	^++^		^++^			
					Rotarod (2x 5 RPM, 2x 3 rpm/5s, 2x 3 rpm/3s	Average trials			^+++^	-		^++^			
Carron et al. (115)	2019	LFP	Moderate	Mixed	Rotarod (1.5 rpm/3s)	% Baseline	T:10; C:10	-	^+++^	-	-				
					Tapered Beam	Ranking		-	^+++^	-	-				
					Tapered Beam	Foot faults		-	^+++^	-	-				
Tan et al. (130)	2020	LFP	Moderate	Mixed	Rotarod (4–40 rpm)	Average 3 trials	T:18; C: 10			-		^+^			
Wright et al. (119)	2017	LFP	Moderate	Mixed	2 cm Beam	Foot faults	T:10; C:10			^+^		^+^			
					2 cm Beam	Traverse time	T:10; C:10			^+^		^+^			
Rowe et al. (111)	2016	LFP	Moderate	Mixed	3 cm Beam	Foot faults	T/C:11–12 2M				^+^	-	-		
							T/C:11–12 4M				-	-			
							T/C:11–12 6M				-				
					3 cm Beam	Traverse Time	T/C:11–12 2M				-	-	-		
							T/C:11–12 4M				-	-			
							T/C:11–12 6M				-				
Sell et al. (81)	2017	LFP	Moderate	Mixed	2.5 cm Beam	Traverse Time	T:37; C:39	-	^+^			-	-		-
					1.75 cm Beam	Ranking		-	^+^			^+^	-		-
Alwis et al. (96)	2012	WD	Mod-Sev	Diffuse	Rotarod (3–30 rpm)	% Baseline	T:19; C: 12	-	^+^	^+^	^+^				
					2 cm Beam	Ranking	T:19; C: 12	-	^+^	^+^	^+^				
Arulsamy et al. (113)	2018	WD	Mod-Sev	Diffuse	Rotarod (3–30 rpm)	Latency to fall	T:6; C: 6			^+++^	-	-			
Albayram et al. (124)	2017	WD	Mod-Sev	Diffuse	0.8 cm beam	Score	T/C = 9–10			^+^			^+^		
					String Suspension (3 trials)	Score				^+^			^+^		
Soblosky et al. (120)	1997	CCI	Mod-Sev	Focal	2.5 cm Beam	Ranking	T:10; C: 10	-	^+^	^+^	^+^	-			
					Pegged 2.5 cm Beam	Foot faults	T:10–13; C:10–14	-		^+^	^+^	-			
Wang et al. (90)	2019	Punch	Mod-Sev	Focal	2 cm Beam	Ranking	T:8; C:8		^++^	^++^	^++^				
Hanscom et al. (116)	2021	CCI	Mod-Sev	Focal	5 mm beam	Foot faults	T/C: 14–21	-	^+++^	^+++^	^+++^				
Vogel et al. (132)	2020	CCI	Mod-Sev	Focal	Rotarod 36 rpm	Latency to fall	T:16; C:16			-			-		
					Rotarod:accelerating		T:20 C:10			-		-			
Cline et al. (31)	2017	CCI	Severe	Focal	2.5 cm Gridwalk	Foot faults	T:15; C:14	-	^+^		^+^				
Xu et al. (121)	2019	CCI	Severe	Focal	2 cm Beam	Traverse Time	T/C: 38		-	^+^	^+^				
He et al. (129)	2020	CCI	Severe	Focal	Rotarod (4–40 rpm)	Average 3 trials	T:11; C: 7				^+^	^+^			
Nissinen et al. (84)	2017	LFP	Severe	Mixed	2 cm Beam	Ranking	T:23; C:10	-	^+++^	^+++^	^++^	-			
Zhang et al. (83)	2005	LFP	Severe	Mixed	Rotating pole (5 rpm)	Ranking	T:14; C: 24				^++^	^++^			
					2 cm Beam	Ranking					^++^	^++^			

^+^*p* < 0.05; ^++^*p* < 0.01; ^+++^*p* < 0.001; -, not significant; Gray, not evaluated. T, TBI; C, control; CCI, controlled cortical impact; CCI-CS, control cortical impact, closed skull; WD, weight drop injury; LFP, lateral fluid percussion.

Following diffuse TBI, moderate-to-severe injury was more likely to lead to acute balance deficits as seen as impaired rotarod performance (96, 113), time to traverse a beam (96), or a score evaluating performance encompassing foot faults or falls (124). In contrast, acute deficits were not seen in most models of mTBI, (93, 106, 131) with only one diffuse mTBI study reporting acute deficits (< 72 h) on the rotarod (97), with these deficits persisting to 1 month following injury and resolving by 3 months (98). Nonetheless following diffuse injury, the overall consensus was that no chronic deficits were seen on the rotarod, regardless of injury severity, from 3 to 24 months post-injury (35, 36, 88, 98, 103, 106, 113). In fact, only a single study found long-term impairment on the rotarod following either mild or moderate diffuse TBI (131). In the mild diffuse TBI group, deficits emerged at 8 weeks following injury and persisted to 18 weeks, whereas with a moderate injury deficits emerged at 4 weeks and similarly persisted to 18 weeks, the latest time point examined (131). The use of other balance tests did detect balance deficits up to 2 months post-injury following mTBI on both time to traverse a 0.5 cm beam (117) and increased missteps on the Erasmus ladder (93). Furthermore, following mod-sev diffuse TBI, worse performance was noted both on an 0.8 cm beam, with performance scored from 0 to 3 depending on how mice were able to traverse the beam and number of falls and foot faults, and on a string suspension assay (124). However, this has not been consistently reported, with other studies investigating mild (80, 103) and moderate-to-severe diffuse TBI (96) showing no deficits when traversing larger beams (0.65–2 cm) (96, 103) or on forelimb placement in the ladder task (80) up to 3 months post-injury, indicating task-dependent effects and that more difficult tasks are required to detect subtle motor deficits.

Compared to diffuse injury, the focal injury was more likely to cause chronic balance deficits. Following moderate focal injury, deficits on the grid walk, balance beam, and rotarod tasks were noted to be 3 months post-injury (28, 103, 120–122, 128, 133), the latest time point assessed. Differing results were seen on the pole test, with an increase in time to turn only emerging at 4.5 months post-injury, with recovery by 6.5 months, which persisted to 9 months post-injury (109). These results were supported by studies in moderate–severe focal TBI, where Hanscom et al. found significantly increased foot faults on the ledged beam from 1 day to 2 months post-injury (116), and a focal punch injury similarly resulted in impaired beam performance up to 6 weeks post-injury (90). With a severe focal injury, balance deficits were consistently noted up to 10 weeks post-injury, the latest time point assessed on the balance beam, rotarod, cylinder test, and grid walk tasks (31, 94, 121, 129). In contrast, two studies did not report chronic balance deficits following moderate–severe focal TBI, although these used a larger beam (118) and altered rotarod parameters (mice were placed on the rotarod already spinning at 36 RPM, rather than gradually increasing speed from 3 RPM) (132). The larger beam would have reduced the complexity of the task, whereas the increased rotarod starting speed may have made the task too difficult for the shams, masking any injury effect.

With a mixed focal and diffuse injury via LFP, mixed results for balance and coordination were seen with a moderate injury. Wright et al. noted increased foot faults and decreased time to cross the beam (119), and Tan et al. found impaired rotarod performance at 3 months post-injury (130). Rowe et al. found a similar pattern in animals injured at 2 months of age, with increased foot faults and time to cross the beam at both 1 and 3 months post-injury but interestingly not in animals injured at 4 or 6 months (111). In contrast, Carron et al. noted acute deficits in performance on the rotarod and tapered ledged beam, which had recovered by 1 week following injury, with no further deficits seen to 2 months post-injury (115). However, with more severe injury, LFP resulted in impaired performance on both the balance beam and rotating pole tasks out to 4 months post-injury, the latest time point examined (83, 84).

In models of repeated mTBI, a higher number of injuries or a shorter interval between injuries were generally associated with more persistent balance deficits. Following 5–7 injuries, chronic deficits on the balance beam, string suspension, and rotarod were noted up to 12 months post-injury (100, 104, 124, 125), although no deficits were seen by 24 months post-injury (36). Only the Mannix et al. (125, 126) and the Mouzon et al. (35) studies failed to find chronic balance deficits following 4–7 impacts. Following three injuries with a 24-h interval between injuries, increased latency to fall on the rotarod was seen up to 6 months post-injury (99, 105), with recovery by 12 months (105). Extending the interval between injuries to 5 days meant that three injuries no longer led to deficits on the rotarod up to 3 months post-injury (106). Interestingly, unlike the rotarod, no deficits were noted on forelimb placement in the ladder walk (80) nor in pole climbing time (99) with 3 injuries, 24 h apart. With two injuries spaced 24 h apart, the number of foot faults on a rotating pole was increased at 3 days post-injury, before returning to sham levels from 7–28 days, before a deficit re-emerged at 2 months following injury (79). By increasing the interval between the two injuries to 3 days, deficits were no longer noted on the balance beam, rotarod, or grid walk tasks either acutely or chronically up to 2 months post-injury (103).

Finally, neither single nor repeated blast injury was sufficient to produce persistent motor deficits at 6 months post-injury, regardless of initial injury severity. With a mild blast injury at 19psi, a single injury led to no balance deficits on the rotating pole or rotarod task to 6 months post-injury (114). In contrast, when two 19 PSI injuries were delivered within 2 min, balance deficits emerged at 6 days, persisted to 4 weeks, with recovery and no further impairment noted following this time point up to 6 months post-injury (114). Similarly, following a single moderate blast impact (50 PSI), acute deficits in latency to fall on the rotarod were noted, which had recovered by 6.5 weeks post-injury (65).

### 3.5. Clinical studies

#### 3.5.1. Motor function test

Overall long-term motor function following TBI was assessed in a single study using the Unified Parkinson's Disease Rating Scale (UPDRS) Motor Examination, which was used to calculate a modified UPDRS (mUPDRS) global motor score, as well as four domain scores: tremor, rigidity, bradykinesia, and posture/gait (40). In retired military veterans (M: 76.4 ± 10.0 years of age) who self-reported TBI [median TBIs = 2 (1.2), 53.2 ± 18.1 years since first TBI, 37.0 ± 22.5 years ago since last TBI], those with a history of moderate–severe, but not mTBI, had a significantly worse mUPDRS global motor score, as well as a worsened score for posture/gait, but not for tremor, rigidity, or bradykinesia, compared to those without a history of TBI (M: 79.4 ± 8.2 years of age) (40) (Table 7).

**Table 7:** Clinical neuroscore evaluation.

**References**	**Year**	**Severity**	**Population**	**Sample size**	**Age (mean)**	**Sex %male**	**Motor test**	** < 5 yrs**	**6–10 yrs**	**11–25 yrs**	**>25yrs**
Gardner et al. (40)	2017	Rep-Mild	Military veterans	T:31–34C:65–68	T: 79.4 C: 76.4	T:82.4%C:94.9%	mUPDRS Global Score				-
							mUPDRS Tremor Score				-
							mUPDRS Rigidity Score				-
							mUPDRS Bradykinesia Score				-
							mUPDRS Posture/Gait Score				-
		Moderate-Severe		T:20 C:65–68			mUPDRS Global Score				^+^
							mUPDRS Tremor Score				-
							mUPDRS Rigidity Score				-
							mUPDRS Bradykinesia Score				-
							mUPDRS Posture/Gait Score				^+^

^+^*p* < 0.05; -, not significant; gray, not evaluated.

#### 3.5.2. Grip strength and fine motor control

Six studies identified herein (19, 25, 26, 51, 53, 76) evaluated chronic alterations in grip strength or fine motor control (Table 8). Grip strength was assessed only in one study comparing individuals with a history of TBI 1–26 years earlier to healthy controls. No differences were seen following either mild or moderate–severe TBI, in either the dominant or non-dominant hand, although the mTBI group was significantly more variable than healthy controls across 10 trials (51). This study also investigated finger dexterity as time taken to touch each of their fingers to their thumb three times. The moderate–severe TBI group at 12.2 years post-injury (range 1–25), but not the mild TBI group at 7.1 years post-injury (range 1–27), had slower finger dexterity in both the non-dominant and dominant hands over 10 trials compared to healthy controls (51). This was not related to age, given that both groups had a similar mean age (35.4 vs. 37.6 years). Similarly, no effects of at least one mTBI (range 1–12, median = 2) in military veterans at a median of 8 years post the most recent TBI were noted in the grooved pegboard task, where pins must be manipulated and rotated to fit a hole (53). With a higher number of repeated concussions and a longer time-period post-injury, however, deficits in fine motor control were seen chronically. Retired rugby league players (mean 8.5 concussions) at almost 20 years post-injury took longer in the O'Connor Finger Dexterity Test, where the time taken to place pegs in holes is recorded compared to controls (26), with similar findings in amateur Australian football players (mean 3.2 concussion) at 22.12 ± 6.73 years following their last injury on the same task (25). Similarly, chronic, but not acute, deficits were seen in a RAM task consisting of rapid wrist supination–pronation movements. Significantly lower movement velocity was found in athletes who sustained their last concussion 30 years earlier (range 27–41 years) (76) but not in those who had sustained their last concussion only 9–34 months earlier (19). Importantly, both groups had the same range of 1–5 concussions, suggesting that these effects may be due to time elapsed since injury, rather than number of injuries.

**Table 8:** Clinical fine motor evaluation.

**References**	**Year**	**Severity**	**Population**	**Sample size**	**Age**	**Sex** **%male**	**Motor test**	**Parameters**	** < 5 yrs**	**6–10 yrs**	**11–25 yrs**	**>25 yrs**
Burton et al. (51)	2002	Mild	Community	T:19; C:26	T:35.36 C: 32.77	T:78.9 C:46.15	Fine motor	Touch fingers to thumb		-		
							Grip strength	Dynamometer		-		
Walker et al. (52)	2018	Combination	Military veterans	T:380; C 73	T:36 C:40.5	T:88% C:79.5%	Fine motor	Grooved Pegboard		-		
De Beaumont et al. (19)	2011	Combination	College Athletes	T:21; C:15	T/C: 22.3	T/C: 100%	Rapid alternating movement Wrist supination-pronation	Velocity	^**+**^ (↑)			
								Sharpness	-			
								Bimanual co-ordination	-			
De Beaumont et al. (76)	2009	Combination	College Athletes	T:19; C:21	T:61; C:59	Not stated	Rapid alternating movement Wrist supination-pronation	Duration				-
								Range				-
								Sharpness				-
								Velocity				^**+**^
Pearce et al. (25)	2014	Rep-Mild	Professional Athletes	T:40; C:20	T:49.3 C:47.6	T/C: 100%	Fine motor	O'Connor Finger Dexterity Test			^**+**^	
Pearce et al. (26)	2018	Rep-Mild	Professional Athletes	T:25; C:25	T:48.4 C:48.8	T/C: 100%	Fine motor	O'Connor Finger Dexterity Test			^**+**^	
Burton et al. (51)	2002	Mod-Sev	Community	T:9; C:26	T:37.56 C: 32.77	T:66.67% C:46.15	Fine motor	Touch fingers to thumb			^**+**^	
							Grip strength	Dynamometer			–	

^+^*p* < 0.05; -, not significant; gray, not evaluated; ↑, increased.

#### 3.5.3. Gait

Several characteristics can be used to assess gait, including spatiotemporal factors, such as cadence, stride length and single and double support time, kinematics in regard to the motion of joints, and kinetics to describe the measurement of the forces required to make a movement (22). Clinical studies varied widely in regards to the gait parameters examined, the tests employed, and equipment used (Table 9).

**Table 9:** Clinical gait studies.

**References**	**Year**	**Severity**	**Population**	**Sample size**	**Age (mean)**	**Sex (%male)**	**Procedure**	**Equipment**	**Measure**	** < 5 yrs**	**6–10 yrs**	**11–25 yrs**	**>25 yrs**
Burton et al. (51)	2002	Mild	Community	T:19; C:26	T:35.36 C:32.77	T:78.9 C:46.15	Turn 360°	None	Speed		-		
							Timed 4 m walk	None	Speed		-		
Vanderploeg et al. (55)	2007	Mild	Military veterans	T:254; C:3214 MVA:539	T:37.8 C:38.5 MVA:37.8	100%	Heel toe walk	None	Abnormal/normal			3x	
Stuart et al. (75)	2020	Mild	Community Self-reported balance instability > 3 months post-injury	T:52; C:59	T:39.6 C:37.0	T:30.8% C:42.4%	13 m walkway, 2 mins	Five sensors strapped to feet, L5, sternum and head	Stride length	^**^			
									Speed	^***^			
									Foot stride angle	^**^			
									Toe off angle	-			
									Single support time	^**^			
									Double support time	^**^			
									Stride time	^**^			
									Turn duration	^**^			
									Turn step number	^*^			
									Turn velocity	^***^			
Martini et al. (54)	2021	Combination	Community Symptoms persisting > 3 months following TBI	T:65; C:57	T:39.6 C:36.9	T:64% C:55%	208 m walk	Five sensors strapped to feet, L5, sternum and head	Speed	^**+**^			-
									Variability	-			
									Rhythm	-			
									Turning	^**+**^			
							Dual task Above ^+^ auditory Stroop		Speed	^**+**^			
									Variability	-			
									Rhythm	^**+**^			
									Turning	^**+**^			
Walker et al. (52)	2018	Combination	Military veterans	T:258;C:47	T:36 C:40.5	T:88% C:79.5%	4 m walk	None	Speed			-	
Martini et al. (66)	2011	Combination	College students	T:25; C:25	T:21 C:20.7	T:60.7% C:50%	4 m walk	GAITr walkway	Speed		^**+**^		
									Double Stance support		^**+**^		
							4 m walk ^+^ obstacle		Speed		-		
									Double Stance support		_		
							Brooks Spatial Memory task ^+^ 4 m walk		Speed		-		
									Double Stance support		-		
							Brooks Spatial Memory task ^+^ 4 m walk ^+^ obstacle		Speed		-		
									Double Stance support		^**+**^		
Pitt et al. (56)	2020	Combination	Military veterans with chronic symptoms	T:8; C:8	T:32.5 C:33.3	T:87.5% C:87.5%	10 m walk, 8–10 trials	Whole body retroreflective marker set with 12 motion analysis system	Speed	-			
									Step length	-			
									Step width	-			
									Medial-lateral COM displacement	^**+**^			
									Peak medial-lateral COM velocity	-			
							Above ^+^ auditory Stroop task		Speed	-			
									Step length	-			
									Step width	-			
									Medial-lateral COM displacement	^**+**^			
									Peak medial-lateral COM velocity	-			
Burton et al. (51)	2002	Mod-Sev	Community	T:9; C:26	T:37.56 C:32.77	T:66.67% C:46.15	Turn 360°	None	Speed		-		
							Timed 4 m walk	None	Speed		-		
Useros Olmo et al. (57)	2020	Mod-Sev	Hospital	T:20; C:19	T:36.1 C:38.2	T:85% C:89.5%	Treadmill 3 kms/hr,	Gait Trainer2	Cadence	-			
							Treadmill 3 kms/hr ^+^ cognitive task			-			
Vasudevan et al. (58)	2014	Mod-Sev	Community	T14; C:11	T:29.7 C:31.1	T:71.4% C:81.8%	Split treadmill: same speed	Markers on toe, ankle, knee, hip, pelvis, shoulder	Stride length	-			
									Stance time	-			
									Step symmetry	-			
									Center of oscillation	-			
									Temporal coordination	-			
							Split treadmill: different speed		Stride length	-			
									Stance time	-			
									Step symmetry	^**+**^			
									Center of oscillation	-			
									Temporal coordination	-			
							Split treadmill: same speed: Post-adaption		Stride length	-			
									Stance time	-			
									Symmetry	-			
									Center of oscillation	-			
									Temporal coordination	-			
Buster et al. (77)	2013	Severe	Community	T:10; C:10	T:36: C:34	Not stated	Elliptical trainer, comfortable stride length, 3 mins	Reflective markers on the pelvis, hip, knee, ankle and foot	Speed		-		
									Cadence		-		
									Stride length		-		
									Motion profile		-		
									Joint angles		^**+**^		
							Treadmill, comfortable speed 3 mins		Speed		-		
									Cadence		-		
									Stride length		-		
									Motion profile		-		
									Joint angles		-		
Williams et al. (59)	2009	Severe	Hospital Able to walk independently 20 m	T:41; C:25	T:29.1 C:27.8	T:75.6% C:64%	12 m walk: Self-selected speed	25 reflective markers on pelvis and lower limbs	Double support		^**+**^		
									Speed		^**+**^		
									Cadence		^**+**^		
									Stride length		^**+**^		
									Stance duration		^**+**^		
									Base of support		^**+**^		
				T:41; C:15			12 m walk: Matched speed		Trunk angle		^**+**^		
									Pelvic angle		-		
									Hip angle		-		
									Knee angle		^**+**^		
									Ankle angle		-		
									Lateral COM displacement		^**+**^		
Williams et al. (22)	2010	Severe	Hospital Able to walk independently 20 m	T:55; C:10	T:28.5 C:27.3	T:72.7% C:50%	12 m walk: Matched speed	25 reflective markers on pelvis and lower limbs	Speed		-		
									Cadence		-		
									Stride length		^**+**^		
									Stance time		-		
									Double support time		-		
									Lateral COM displacement		^**+**^		
									Peak ankle power		-		
									Peak hip power (initial)		^**+**^		
									Peak hip power (prewswing)		^**+**^		
				T:36;C:10	T:27.3 C:27.3	T:77.8% C:50%	12 m walk: Fastest speed		Speed		-		
									Cadence		-		
									Stride length		-		
									Stance time		-		
									Double support time		-		
									Lateral COM displacement		^**+**^		
									Peak ankle power		^**+**^		
									Peak hip power (initial)		^**+**^		
									Peak hip power (prewswing)		-		
Williams et al. (23)	2013	Severe	Hospital Able to run independently 20 m	T:44; C:15	T:27.9 C:28.1	T:81.8% C:73.3%	15 m run: Matched speed	25 reflective markers on pelvis and lower limbs	Speed	-			
									Cadence	^**+**^			
									Stride length	^**+**^			
									Stance time	^**+**^			
									Flight phase	^**+**^			
									Base of support	-			
									Trunk flexion	-			
									Pelvic rotation	^**+**^			
									Hip extension/adduction	-			
									Knee flexion	^**+**^			
									Ankle flexion	-			
									Lateral COM displacement	^**+**^			
									Ankle power	^**+**^			
									Knee power	^**+**^			
									Hip power	^**+**^			
							15 m run: Fastest speed		Speed	^**+**^			
									Cadence	^**+**^			
									Stride length	^**+**^			
									Stance time	-			
									Flight phase	^**+**^			
									Base of support	^**+**^			
Williams et al. (24)	2016	Severe	Hospital Able to walk independently 20m	T:35; C:25	T:28.4 C:27.8	T:74.3% C:64%	12 m walkway: matched speed	25 reflective markers on pelvis and lower limbs	Hip work	-			
									Knee work	-			
									Ankle work	^**+**^			
									Hip power	-			
									Knee power	-			
									Ankle power	^**+**^			

^*^p < 0.05, significant; ^***^strong power; ^**^medium power; ^*^low power; -, non-significant; gray, not assessed.

No difference in gait speed over a distance of 3–4 m was seen either in a group of military veterans (53) or in a cohort recruited from the general population (51) on average a decade following their last injury. Conversely, more sophisticated analysis employing an 8-m electronic walkway found that students with a history of concussion with a mean time since injury of 6.32 years had greater time in double-leg stance support and less time in single-leg stance support, throughout the gait cycle (66). The more difficult task of heel to toe walking was also found to be affected by previous mTBI, with veterans with a history of mTBI approximately 16 years ago being three times as likely as normal controls to have their performance ranked as abnormal by a neurologist (55). Other studies investigating mTBI specifically recruited patient populations with persistent symptoms. Stuart et al. aimed to develop a model to describe differences in gait seen in individuals with a history of mTBI sustained approximately 18 months ago (median 551 days) who had self-reported balance instability. Participants walked over a 13-m distance for 2 min with inertial sensors detecting gait. Differences in the mTBI cohort compared to healthy controls related to increased variability, decreased rhythm, and reduced pace in parameters such as stride length and time, alongside increased turn duration and velocity (75). These results were only partially supported by another study, which recruited individuals with a history of mTBI with symptoms persisting >3 months but did not require these symptoms to be specifically balance related. Participants were on average a year from injury, with statistically significant differences only detected in pace and turning, but not in rhythm and variability, over a ~200-m walk with multiple 180° turns (124). It should be noted that the Stuart et al. study did not report *p-*values but rather investigated effect size only, which could also account for the differences between these studies. Conversely, a much smaller study (*n* = 16) of symptomatic veterans with a history of mTBI 3.5 ± 1.7 years previously found no difference in gait speed or stride length over 10 m (33). Given that the Stuart et al. study included 111 participants (75) and the Martini et al. study 68 (124), any differences may relate to the small sample size.

Indeed, in comparison with symptomatic mTBI, moderate–severe TBI at least 18 months earlier (mean: 35.5 ± 20.2 months) found no difference in cadence on a treadmill at 3 km/h for 2 min (77). Similar findings were found more chronically, with a moderate TBI (range 1–26 years prior) not producing deficits in walking or turning speed on a walkway (51) or treadmill or elliptical trainer task (77). With a more difficult task, participants were placed on split-belt treadmill, such that the speed required for each leg could be varied (58). Those with a history of moderate–severe injury an average of 2.9 ± 1.7 years prior took longer to adapt to the belts being at different speeds, seen as an decrease in step symmetry, but were no different in the baseline task or post-adaptation when the two belts were at the same speed (58).

Self-selected walking (22) and running (23) speeds were slower in individuals with a previous history of severe TBI an average of 5–6 years earlier, with participants chosen for their ability to walk and run independently, respectively, while still attending physiotherapy for mobility limitations. When healthy controls matched these speeds, no difference in either cadence or stance time were seen with walking (22), whereas, with running, a previous history of TBI led to increased cadence and shorter stride length to produce the same speed (23). In the only study which did not report a difference in walking speed, 19 of 52 participants were unable to walk at the faster speed, negating the measurement (22). Nonetheless, numerous kinetic and kinematic alterations were associated with both running and walking following severe TBI, including alterations in ankle power generation (22, 24) and knee stability (23), which likely drive these alterations in gait. In a follow-up study, these authors followed patients for 6 months following severe TBI an average of 2 years earlier with access to a rehabilitation program (83). At baseline, previous TBI was again associated with significantly slower self-selected walking speed than healthy controls (83). However, at the 6-month follow-up, walking speed had significantly improved, such that there was no longer any difference compared to healthy controls, with an associated improvement in ankle power generation, indicating that these deficits can improve (83).

In addition to assessing gait alone, several studies investigated the effect of increasing difficulty via the inclusion of obstacles (54) and/or cognitive tasks (54, 56, 66) on gait following mild TBI (combined single and multiple). In symptomatic individuals < 5 years following the last injury, adding an auditory Stroop task had little effect on gait (54, 56). In a community cohort, changes in rhythm were seen in the dual vs. single task in those with a history of mTBI compared to controls (54), whereas in a small cohort of military veterans no additive effect was seen with alterations in center of mass displacement seen in both walking alone and the dual task (56). Adding obstacles or a spatial memory task actually reduced the performance in healthy controls, such that differences seen in speed and double stance support time with walking alone compared to those with a history of TBI were no longer present (66). Indeed, only combining the memory task with obstacles while walking re-introduced the increase in double stance support time in those with a history of mTBI 6 years earlier (66). Hence, abnormalities in gait following mTBI may be detected by single task alone, without requiring the more difficult combined tasks.

#### 3.5.4. Posture and balance

The effects of a prior TBI on chronic alterations in static balance were assessed under several conditions, with the majority of studies only investigating 1–5 years post-injury (52, 53, 57, 60, 63, 64, 67–69, 74, 78, 134) and no studies looking at greater than 10 years post-injury (Table 10). In healthy populations, maintenance of postural stability does not require large amount of conscious effort and is regulated by subconscious reflexive actions of the CNS to interpret and act in accordance to perceived sensory feedback information from the visual, somatosensory, and vestibular systems (135). Manipulating the type and/or amount of information being processed by these three sensory feedback systems increases the difficulty of balance tasks and can reveal injury effects (136–138). Across the included studies, postural control was examined primarily by evaluating alterations in center of mass while standing, with concomitant manipulation of the sensory information available via altering the standing surface, closing eyes, or altering the visual surroundings. Studies also incorporated the effects on balance in functional reaching involving either the leg (60) or arm (67, 119) as well as the effects of bimanual lifting of weights (64).

**Table 10:** Clinical postural stability studies.

**References**	**Year**	**Severity**	**Population**	**Sample size**	**Age**	**Sex (%male)**	**Motor test**		**Method**	**Parameters**		** < 5 yrs**	**6–10 yrs**	**11–25 yrs**	**>25 yrs**
Geurts et al. (74)	1999	Mild	Hospital Persistent symptoms	T:15;C:20	T:35.9 C:35.4	T:53.3% C:60%	Postural control: Eyes open, closed or with simple cognitive task		Balance board 2x 30 secs per variable	**Anteroposterior sway**		^**+**^			
										**Lateral sway**		^**+**^			
										**Weight shifting**		^**+**^			
Wright et al. (69)	2018	Mild	Military veterans	T:9;C:19	T:25.95 C:33.57	T/C: 45%	Postural control Firm or foam, eyes open or closed, static or rolling scene		Virtual environment TBI screening 3 × 30secs per variable	Sway area		-			
Pan et al. (65)	2015	Mild	Military veterans Non-symptomatic	T:6, C:10	T:26.5 C: Not stated	T:100% C:Not stated	Postural control: On floor or foam; Eyes open or closed		Markers on sacrum, pelvis, C7 and shoulder.	Pelvic postural sway		-			
										Pelvic sway path length		-			
										Pitch trunk angle		-			
										Roll Trunk angle		-			
							Postural control^**+**^ perturbation: On floor or foam; Eyes open or closed			Upper trunk sway path length		-			
										Pelvic sway path length		-			
										Oscillations		-			
			Military veterans Symptomatic	T:8; C:10		T:87.5% C:Not stated	Postural control: On floor or foam; Eyes open or closed			**Pelvic postural sway**		^**+**^			
										**Pelvic sway path length**		^**+**^			
										**Pitch trunk angle**		^**+**^			
										**Roll trunk angle**		^**+**^			
							Postural control^**+**^ perturbation: On floor or foam; Eyes open or closed			**Upper trunk sway path length**		^**+**^			
										Pelvic sway path length		-			
										**Oscillations**		^**+**^			
Rosenblum et al. (68)	2020	Mild	College Athletes	T:91;C:129	T:18.9; C:19.1	T:56.6% C:56.6%	Sensory organization test Fixed/ sway surface, eyes open/closed, surrounding/sway-referenced surrounding,		Smart Balance Master System 3 × 20secs per variable	Anterior-posterior sway (equilibrium score)		-			
										Somatosensory sensory ratio		-			
										Visual sensory ratio		-			
										Vestibular sensory ratio		-			
Ustinova (73)	2017	Mild	College Athletes	T:13; C:13	T:34.9 C:33.8	T:30.8% C:38.4%	Functional reach Interception of targets via arm at 5 fixed location		3D video game 30 reflective body markers 10 × 90sec games	Postural angular displacement			-		
										**Postural angular velocity**			^**+**^		
										Arm angular displacement			-		
										**Arm angular velocity**			^**+**^		
Wright et al. (69)	2018	Combination	Military veterans	T:14; C:19	T:25.95 C:33.57	T/C: 45%	Postural control Firm or foam, eyes open or closed, static or rolling scene		Virtual environment TBI screening 3 × 30secs per variable	Sway area		^**+**^			
Helmich et al. (78)	2016	Combination	University students w/o symptoms	T:13; C:10	T:29 C:27	T:Not stated C:40%	Postural control Stable vs. unstable surface, eyes open, closed or blurred		Force platform 10 × 10sec trials per variable	COP path		-			
										COP area		-			
										Effort of balance		-			
							Balance Error Scoring System			Score		-			
			University students with symptoms	T:7; C:10	T:26 C:26	T:Not stated C:40%	Postural control Stable vs. unstable surface, eyes open, closed or blurred			COP path		-			
										COP area		-			
										**Effort of balance**		^**+**^			
							Balance Error Scoring System			**Score**		^**+**^			
Degani et al. (72)	2017	Combination	Community	T:11; C:11	T: 29.4; C: 26.8	T:45.5% C:36%	Postural control Natural stance vs crossed arms		Force platform 10 mins	**Body sway area**		^**+**^			
										**Amplitude of COP displacement**		^**+**^			
										**Mean velocity of COP displacement**		^**+**^			
										**Frequency of COP displacement**		^**+**^			
										**Regularity of COP displacement**		^**+**^			
De Beaumont et al. (76)	2011	Combination	Collegiate athletes	T:21; C:15	T/C: 22.3	T/C: 100%	Postural control		Force platform 2 × 30 sec trials	**Regularity of COP displacement**		^+^			
										Amplitude of COP displacement		-			
Johnston et al. (60)	2020	Combination	Collegiate athletes	T:30; C:90	T:20.3 C:20.2	T/C: 83.5%	Y Balance test (Stand on one leg, reach other in anterior, posteromedial and posterolateral direction)		Lumbar inertial sensor 3 trials	Anterior	Reach distance	-			
											Regularity	-			
											Amplitude	-			
										Posteromedial	Reach distance	-			
											Regularity	-			
											Amplitude	-			
										Posterolateral	Reach distance	-			
											Regularity	-			
											Amplitude	-			
Ledwidge et al. (134)	2020	Combination	Collegiate athletes	T:21; C:24	T:20.17 C:20.03	T:90% C:79%	BESS balance test Feet together, non-dominant only, tandem on firm or foam surface		-	Score		-			
Lee et al. (71)	2020	Combination	College students	T:11; C:14	T:28.7 C:22	T:52% C:35.7	Postural stability		Force platform 120 sec	Body sway area			-		
										Amplitude			-		
										Mean velocity			-		
										Frequency			-		
										Regularity			-		
										Asynchrony AP and ML			-		
Reilly et al. (70)	2020	Combination	Community	T:27; C:27	T:26.1 C:28.6	T:44.4% C:77.8%	Postural stability	Bipedal only	Force platform	Mean velocity			-		
										Path length			-		
										AP sway			-		
										ML sway			-		
										Body sway area			-		
										Regularity (AP)			-		
										Regularity (ML)			-		
								Bipedal ^+^ cog task		Mean velocity			-		
										Path length			-		
										**AP sway**			^**+**^		
										ML sway			-		
										**Body sway area**			^**+**^		
										**Regularity (AP)**			^**+**^		
										Regularity (ML)			-		
								Unipedal only		Mean velocity			-		
										Path length			-		
										AP sway			-		
										ML sway			-		
										Body sway area			-		
										**Regularity (AP)**			^**+**^		
										**Regularity (ML)**			^**+**^		
								Unipedal ^+^ cog task		Mean velocity			-		
										Path length			-		
										AP sway			-		
										**ML sway**			^**+**^		
										**Body sway area**			^**+**^		
										**Regularity (AP)**			^**+**^		
										**Regularity (ML)**			^**+**^		
Rosenblum et al. (68)	2020	Combination	College Athletes	T177;C:129	T:19.1 C:19.1	T:57.6% C:56.6%	Sensory organization test Fixed/ sway surface, eyes open/closed, surrounding/sway-referenced surrounding,		Smart Balance Master System 3 × 20secs per variable	Anterior-posterior sway (equilibrium score)		-			
										Somatosensory sensory ratio		-			
										Visual sensory ratio		-			
										Vestibular sensory ratio		-			
Sosnoff et al. (63)	2011	Combination	College athletes	T:62; C:162	T/C: 20.04	T/C: 67.8%	Sensory organization test Fixed/ sway surface, eyes open/closed, surrounding/sway-referenced surrounding		NeuroCom Smart Balance Master 3 × each test	Composite balance score		-			
										Somatosensory sensory ratio		-			
										Visual sensory ratio		-			
										Vestibular sensory ratio		-			
										**Regularity (AP)**		^**+**^			
										**Regularity (ML)**		^**+**^			
Walker et al. (52)	2018	Combination	Military veterans	T:248; C:47	T:36 C:40.5	T:88% C:79.5%	Sensory organization test	Eyes open/fixed surface	NeuroCom Smart Balance Master 3 × each test	Equilibrium score			-		
								Eyes closed/fixed surface		**Equilibrium score**			^+^		
								Eyes open/fixed surface/sway surroundings		**Equilibrium score**			^+^		
								Eyes open, sway surface, fixed surroundings		Equilibrium score			-		
								Eyes closed, sway surrounding		Equilibrium score			-		
								Eyes open, sway surface, sway surrounding		Equilibrium score			-		
								Composite score		**Equilibrium score**			^+^		
Walker et al. (52)	2018	Combination	Military veterans	T:414; C:78	T:36 C:40.5	T:88.2% C:79.5%	Sensory organization test	Eyes open/fixed surface		Equilibrium score			-		
								Eyes closed/fixed surface		**Equilibrium score**			^**+**^		
								Eyes open/fixed surface/sway surroundings		**Equilibrium score**			^**+**^		
								Eyes open, sway surface, fixed surroundings		Equilibrium score					
								Eyes closed, sway surrounding		Equilibrium score					
								Eyes open, sway surface, sway surrounding		Equilibrium score					
Wright et al. (69)	2018	rmTBI	Military veterans	T:5;C:19	T:25.95 C:33.57	T/C: 45%	Postural control Firm or foam, eyes open or closed, static or rolling scene		Virtual environment TBI screening 3 × 30secs per variable	**Sway area**		^**+**^			
Rosenblum et al. (68)	2020	rmTBI	College Athletes	2^*^TBI:52 3^*^TBI:34; C: 129	2^*^T:19.1 3^*^T:19.8 C:18.1	2^*^T:62% 3^*^T:52% C:56.6%	Sensory organization test Fixed/ sway surface, eyes open/closed, surrounding/sway-referenced surrounding,		Smart Balance Master System 3 × 20secs per variable	Anterior-posterior sway (equilibrium score)		-			
										Somatosensory sensory ratio		-			
										Visual sensory ratio		-			
										Vestibular sensory ratio		-			
Walker et al. (52)	2018	rmTBI	Military veterans	T:248; C:47	T:41 C:46	T:88.3% C:78.7%	Sensory organization test	Eyes open/fixed surface	NeuroCom Smart Balance Master 3 × each test	Equilibrium score			-		
								Eyes closed/fixed surface		**Equilibrium score**			^**+**^		
								Eyes open/fixed surface/sway surroundings		**Equilibrium score**			^**+**^		
								Eyes open, sway surface, fixed surroundings		**Equilibrium score**			^**+**^		
								Eyes closed, sway surface		**Equilibrium score**			^**+**^		
								Eyes open, sway surface, sway surrounding		Equilibrium score			-		
								Composite score		**Composite Equilibrium Score**			^**+**^		
Useros Olmo et al. (57)	2020	Mod-sev	Hospital	T:20; C:19	T:36.1 C:38.2	T:85% C:89.5%	Postural control	Standing only	Force platform 2 mins recording	Displacement of COP		^**+**^			
								Standing ^+^ numerical cog task				+			
								Standing ^+^ spatial memory cog task				+			
Zhang et al. (83)	2002	Mod-sev	Rehabilitation	T:10; C:10	T:30.6 C:31.8	T:50% C:50%	Functional reach	Unpredictable	Force platform Board with two lights	**Stability ratio**		^**^			
								L predictable				^**^			
								R predictable				^***^			
Arce	2004	Severe	Community	T:7; C:10	T:26.4 C: 24.9	T:100% C:100%	Postural control: Bimanual lifting	No load × 3	Tetrax posturography system- two force platforms	**Stability score: standing**		^**+**^			
								4kg × 6		Forward weight shift		-			
								8kg × 6		% change vertical ground reaction force		-			
Buster et al. (77)	2013	Severe	Community	T;10: C:10	T:36; C:24	Not stated	Berg Balance test		None	**Score**			^**+**^		
							Dynamic posturography		Tilt platform 3 × 120 secs	**Dynamic movement analysis score**			^**+**^		

^+^*p* < 0.05, -, not significant, ^***^strong power, ^**^medium power.

A history of asymptomatic mTBI < 5 years earlier had no effects on postural control while standing, regardless of alterations in the support surface or visual feedback in a variety of cohorts including military veterans (65, 119), college athletes (68), and university students (78). This included measures of the center of pressure sway area (69), sway path length (65), trunk pitch and roll angle (65), and the degree of anterior–posterior sway (68). On the one hand, in an arm functional reach task, mTBI, an average of 5.8 years earlier, was associated with reduced postural angular velocity, although no changes in angular displacement were noted (73). On the other hand, symptomatic mTBI was associated with greater alterations in balance, with increased postural sway both during quiet standing (65, 74) and when suddenly perturbed (65).

Repeat mild injury, either investigated as a separate cohort (68, 69) or via combining those with a history of single and repeated injuries (60, 63, 68–72, 76, 134), had more mixed effects on balance. In general, more difficult tasks were required to detect differences between those with a history of mTBI (1 or more) and healthy controls (52, 63, 69–71, 78). For example, in college students, no changes in amplitude, velocity, frequency, or regularity were seen in quiet standing in those with a mean time since the last injury of 7.1 years (mean 2.5 injuries) (71). However, two studies were able to detect postural changes with quiet standing alone, with collegiate athletes 19 months since their last injury (range 1–5 injuries) exhibiting an increase in center of mass oscillation irregularity (76) and a cohort recruited from the community with one or more mTBIs also at 19 months post-injury having a larger body sway area, a larger displacement amplitude in the medio-lateral direction, a slower body oscillation in both directions, and a more irregular pattern of body oscillation (72). In contrast, Wright et al. only noted a difference compared to healthy controls in individuals with more than one mTBI with the last injury at least a year ago in the most difficult condition, where participants were required to stand on a foam surface with a dynamic visual surrounding, leading to the increased center of pressure sway area (69). Similarly, Helmich et al. only saw an increase in effort of balance in symptomatic individuals within a mean of 2 years post-injury during balance on an unstable surface, with eyes closed or a combination of both, but not on a stable surface or with eyes open (78). At a year post-injury, Reilly et al. also found no effect of previous mTBI (combined single and multiple) on bipedal or unipedal stance alone but did see an increase in sway and decreased regularity when combined with a cognitive task (70). Conversely, Rosenblum et al. found no differences on the Sensory Organization Test at 2–3 years following last injury in a population of collegiate athletes (68). The Sensory Organization Test evaluates quiet standing under six different conditions (either fixed/sway surface, eyes open/closed, or surrounding normal/sway-referenced), thus involving increasing task difficulty. This was regardless of whether analysis looked at single vs. multiple injuries compared to healthy controls, or when those with a history of single or rmTBI were combined (68). In contrast, the same test in military veterans at an average of 10 years post-injury did find task-specific effects, with a decrease in equilibrium score in the eyes closed/fixed surface and eyes open, fixed surface/sway surroundings conditions only when looking at combined single and rmTBI and in these conditions, alongside the sway surface with eyes open or closed conditions, when analysis investigated the rmTBI cohort separate from single injuries (52, 53). Only one study utilized a different task, the Y Balance Test, where participants stand on one leg and reach the other in an anterior, posteromedial, or posterolateral direction (60). Acute deficits, with an increased amplitude of center of mass in the posteromedial and posterolateral direction in those with a history of TBI a median of 294 days ago, had resolved in those with their last injury a median of 3.5 years ago, with no deficits noted compared to healthy controls (60).

With a more severe injury, the same level of analysis examining multiple parameters such as regularity, amplitude, frequency, and velocity changes in the center of pressure has yet to occur, but consistent alterations in balance have been reported out to 10 years post-injury. A prior moderate–severe TBI a mean of 3 years earlier was associated with the increased center of pressure displacement across three tasks: standing only, standing with a numerical task, and standing with a spatial memory task, with no significant alterations in performance between the different tasks (57). Similarly, a prior severe TBI 10 years earlier led to a decrease in dynamic post-urography scores, where postural changes in response to a tilt platform were examined, alongside a higher Berg balance score (77). A functional reach task where participants were able to sit in a wheelchair while moving their arm to touch a target that appeared in either a predictable or unpredictable fashion found a medium–large effect size of prior moderate–severe TBI on stability ratio during the task (67). In a bimanual lifting task, although a history of previous severe TBI 2–10 years earlier was associated with greater instability in the quiet stance phase, the postural adjustments that occurred to lift 4 or 8 kg weights were not different from that of healthy subjects (64). Thus, balance changes appear to be similarly evident, particularly with quiet standing following more severe injury.

## 4. Discussion

This systematic review investigated chronic motor outcomes following TBI and the effect of injury severity. The results of this study provide a comprehensive overview of the current state of understanding of motor changes following TBI, highlighting limitations and gaps of existing research that are critical to filling in order to suggest guidelines for rehabilitation programs following TBI. There was little consensus across the articles presented, with a wide variety of motor domains examined, as well as significant differences in the methodology of the tests utilized and parameters reported. Indeed, the lack of consensus in the approaches used in assessing and reporting chronic motor outcomes in both preclinical and clinical models of TBI limits the generalizability of the findings. In the future, more standardized testing parameters and protocols for motor tasks would assist in comparing findings. For example, the development of common data elements for both preclinical and clinical studies would be of benefit, given that standardization and harmonization of data collection are of paramount importance (139).

Overall, there was a paucity of clinical studies investigating motor outcomes beyond 10 years post-injury, with only six identified within this review. The majority of these studies investigated fine motor control (25, 26, 51, 76), meaning that the long-term effects of TBI on gross motor functions, such as gait and postural control, have not been extensively studied. Furthermore, there was a lack of longitudinal clinical studies investigating how motor performance changes over time in the same cohort in the chronic phase post-injury. Similarly, in preclinical work, only nine studies investigated to 12 months post-injury (35, 36, 81, 82, 100, 105, 108, 114) and, of these, only two studies to 18–24 months post-injury (35, 36). More chronic studies are, therefore, needed to understand how a history of TBI interacts with normal aging to affect motor performance. Imaging studies have suggested that a history of TBI accelerates the rate of brain atrophy (25, 26, 40, 52, 55, 76) and studies investigating cognition have suggested TBI is associated with an earlier age of cognitive decline, not necessarily associated with a specific neurodegenerative disorder (140). Whether TBI similarly leads to earlier physical decline needs further investigation. This is particularly relevant given the growing literature linking a history of TBI to the later risk of neurodegenerative motor disorder development, particularly motor neuron disease (MND) and PD. For example, Wright et al. reported ALS-like pathological changes, accompanied by persistent motor deficits, at 12 weeks, but not 1 week, following a moderate experimental TBI in rats (141). This suggests that TBI may begin an insidious neurodegenerative process that predisposes an individual to the later development of motor neuron disease. This is in line with several previous studies conducted with professional athletes, including National Football League (NFL), (142, 143) soccer (144–147), and rugby union players (41). Overall, meta-analyses have suggested a 1.3- to 1.7-fold increase in motor neuron disease risk due to a prior history of TBI (38, 148, 149); however, not all literature has been consistent (150). Similar findings have also been reported for PD, with a doubling of deaths due to PD in former professional soccer players compared to a matched control group drawn from the general population (146). Even a mild TBI has been shown to increase risk of PD by 56% in US military veterans, after adjusting for demographics and comorbidities (40). Several potential biological mechanisms have been proposed to explain this link, including chronic neuroinflammation, metabolic dysregulation, and pathological upregulation of several key PD-linked proteins, including alpha-synuclein, hyperphosphorylated tau, amyloid precursor protein, TDP-43 and, more recently, leucine-rich repeat kinase 2 (LRRK2) and its Rab protein substrates [see Delic et al. for review (151)]. Motor dysfunction may also play a role in other neurodegenerative diseases linked to TBI, including chronic traumatic encephalophathy (CTE), which is characterized by the accumulation of hyperphosphorylated tau aggregates (148). Clinical data from 298 donors diagnosed with CTE identified motor symptoms in a large portion of cases, with gait and balance disturbance noted in 51% and signs of parkinsonism in up to 28% of cases (149). Thus, tracking alterations in motor function longitudinally in those with a prior history of TBI may allow for earlier identification, and subsequently treatment, of those at risk for the development of MND, PD, or CTE, currently a major area of clinical need.

Despite this significant gap, key findings from clinical studies conducted to date of chronic motor alterations following TBI suggest that measures of balance, including postural control and gait, could differentiate between levels of injury severity in those who had suffered there injury in the last 10 years and, importantly, could discriminate between symptomatic and asymptomatic mTBI sufferers. Balance requires multiple input and integration centers spanning the entire brain, with damage to any of these structures or their associated white matter networks resulting in balance impairment (136). A key feature of post-concussion syndrome may be disruption of these networks, subtlety impairing balance control. Given that stressing the sensorimotor integration centers of the brain elicited the greatest degree of impairment, it suggests that, following injury, there may be limited access to neural resources capable of compensating for reductions in sensory feedback information (either visual, vestibular, or somatosensory), as opposed to gross decreases in musculoskeletal or aerobic functional capacities (138). Entropy measures of postural sway parameters were particularly shown to be affected by symptomatic mTBI, with these measuring the regularity of center of pressure oscillations (137). From a motor control perspective, more regular values are interpreted as indicating a less stable system, as damage to neural tissue results in a reduced capacity for the complex oscillatory networks within the brain to produce and maintain upright posture under a wider variety of movement patterns (152). Decreased entropy values have been reported acutely following mTBI (153, 154) and are shown here to persist in a subpopulation of symptomatic sufferers. The specific mechanisms driving these balance disruptions, however, require further investigation.

Mechanistic investigations may be limited to date, due to significant differences in the examination of balance in preclinical models compared to measures employed clinically. Relatively few preclinical studies incorporated gait analysis, which could be due to the technology required to perform detailed analysis. Surprisingly, the one consistent finding seen in more severe clinical TBI, a reduction in speed, was not replicated in preclinical studies. Indeed, minimal gait deficits, in general, were found in preclinical studies, with only a reduction in swing speed and stride length at 6 months following moderate focal injury (29, 30) but no deficits at 1 month following a more severe injury (94). This may reflect the differing mechanisms of injury and severity of preclinical compared to clinical models. Preclinical models are limited in their abilities to model more severe TBI, which are associated with prolonged stays within the intensive care unit and long periods of rehabilitation, which may impact upon motor function (155). Furthermore, the location of contusional injuries differs in preclinical compared to clinical models, typically found in the pre-frontal and temporal lobes clinically (156) and the parietal lobes in preclinical models (157). Key differences in gait are also obviously evident in biped vs. quadrupeds, with center of mass higher in bipeds than quadrupeds (158) and increased frequency of gait patterns at higher speeds, such as trotting and galloping, in rodents, which are generally not seen in bipedal human (159). However, there were some preclinical findings that were supported clinically, with a model of mTBI finding alterations in base of support to 3 months post-injury (88), with clinical studies similarly showing an alteration in the equivalent double vs. single-stance support at 6 years post-injury (66). Thus, incorporation of longitudinal gait analysis out to more chronic time points in preclinical models of TBI would be useful.

Furthermore, there are no static tests of balance utilized in preclinical studies. Instead, balance assessment pre-clinically incorporates transitional movements utilizing tasks such as the rotarod, balance beam, grid walk, and ladder walk, which are all scored with gross parameters, such as number of foot faults, latency to cross, and speed achieved on the rotarod. These measures may not be sensitive enough to detect subtle deficits, particularly in models of mTBI, especially given that the read out measures are relatively crude, a limitation that has previously been noted elsewhere (160). Indeed, even in more severe models of diffuse injury, chronic (>6 months) impairments in motor performance were not seen, unlike focal or mixed injury models (30, 100, 122), where more widespread disruption of motor pathways may occur. It has previously been noted that the lack of functional deficits in preclinical models is surprising given the amount of histological damage (161). Refinement of motor tests is needed to discern whether this is because the damage is not sufficient to drive functional changes, or whether the motor tests used are not sensitive enough. For example, utilizing center of pressure measurements may be an option for future studies, with this successfully employed previously in models of vestibular injury in rodents (162), given the sensitivity of the task in clinical work.

Another discrepancy between clinical and preclinical studies is the incorporation of fine-motor specific tasks. Although some of the balance tasks outlined above, such as the grid walk and rotarod, incorporate aspects of fine motor performance, the effects of injury specifically on this domain cannot be discerned. Furthermore, tasks like the adhesive removal test may be complicated by the presence of sensory deficits (163). Notably, given that a history of repeated injury clinically appeared to be associated with poorer performance on dexterity tasks (25, 26), the need for greater inclusion of these within preclinical work is supported. Only two studies incorporated a fine-motor specific task in the pellet reaching (34) and Montoya staircase tasks (94). These were only utilized following moderate–severe and focal injury, noting deficits to 6 weeks post-injury, making comparisons with the clinical work, where moderate–severe injury led to deficits at 10 years post-injury (51), but repeated mild TBI at >20 years, (25, 26) difficult. Investigation of other forepaw dexterity-based tasks, such as the vermicelli or cappelini handling tests (163), would also be useful to add to motor behavioral batteries post-TBI.

Alongside the need to utilize a wider variety of preclinical motor tasks, the field would also benefit from a broadening of the animals used. Preclinical rodent models have some limitations in the ability to fully model the types of white matter damage encountered in the diffuse injury of any severity due to the relative lack of myelinated tracks (164). Indeed, single diffuse or mixed injury models did not produce long-term motor deficits (>6 months) (36, 81, 108, 111, 112) compared to purely focal injury (29, 30), although there were obviously few studies that investigated these chronic time points. Promising gyrencephalic models with more extensive white matter tracts may provide a key bridge between preclinical and clinical work with development of porcine (165), ferret (166), and sheep (164) TBI models that could be utilized for future investigation of longitudinal motor deficits. A number of motor tasks have already been developed for these models including gait analysis (160) which provide useful information about the longitudinal trajectory of motor impairment.

In addition, studies to date have failed to take into consideration what effect age at injury may have on long-term motor outcomes following TBI. Motor function is well known to decline substantially with advancing age, with changes at the level of the motor unit (167), as well as at the neural level [for review, see Seidler et al. (168)]. For example, King et al. have demonstrated that age-related declines in motor performance are associated with stronger internetwork resting-state connectivity, suggesting breakdown of organization of large-scale brain networks (169). Similar changes in resting-state functional connectivity have been noted following TBI (170). It is, therefore, reasonable to hypothesize that advanced age may exacerbate alterations in motor performance following injury. In line with this, older age is known to be associated with poorer outcome following TBI, with older adults having the highest rate of hospitalization and death following TBI (171). Older adults have also been shown to experience greater decline in Disability Rating Scale scores over the first 5 years following injury (172). Despite this, however, to date, no clinical studies have investigated the effect of age at injury on chronic motor outcomes. Pre-clinically, only one study incorporated rats injured at different time points in adulthood, with no effect on overall long-term motor performance noted (111). Future studies should, therefore, be designed to include assessment of how age at time of injury may influence motor response. Indeed, in the preclinical literature, there is a growing call to include aged animals in the modeling of neurological disease more generally (173).

Although the current study was limited to chronic motor outcomes, it should be noted that these do not occur independent of effects on other functional domains, such as cognition. For example, following mTBI, it was found that there was a significant association between cognitive and motor function following injury but not prior (174). A potential explanation is that the effects of injury on attentional networks, which are a driver of cognitive dysfunction (175), also impair performance on numerous motor tasks, including postural control (176), gait (177), and the ability to perform fine motor tasks (178). Executive function more broadly is also key for motor performance, with patients with normal measures of executive function following moderate–severe TBI demonstrating better balance and agility and increased speed of walking and running than those with executive function deficits (179). Further study should, therefore, examine chronic motor outcomes in the context of the effects on broader functional domains, including cognition, in order to better explore the bidirectional nature of these relationships. This is particularly important given that effects on cognitive function can affect ability to participate in physical rehabilitation programs, which can have detrimental consequences for functional motor recovery.

Finally, it should also be noted that a high risk of bias was noted for the majority of preclinical and clinical studies included here, which may have influenced the results. For the preclinical studies, key sources of bias included random outcome assessment and blinding of outcome assessment, as well as allocation concealment. For clinical studies, a key source of bias was blinding of outcome assessment, as well and blinding of participants and personnel. If researchers are not blinded, this will have implicit biases on the data recording process and potentially the randomization of the study and random outcome detection, making it difficult to truly interpret results. Given the high risk of bias and high degree of heterogeneity between studies, it was not feasible to conduct a meta-analysis in the current study; however, in the future, it may be of interest to consider meta-analysis on specific data subsets.

In conclusion, despite the known relationship between mobility and quality of life following TBI (6), chronic motor performance following injury is less well studied than either cognitive or affective outcomes. Additionally, across the few studies conducted, significant differences in experimental paradigms employed in both clinical and preclinical studies make it difficult to discern the pattern of motor deficits in the subacute and chronic phase following injury, with more standardized protocols required. Furthermore, a broadening of the motor batteries utilized within preclinical studies is warranted to more closely mirror the types of balance and fine motor deficits identified clinically. This review highlights the need for more consistent investigation and reporting of long-term motor deficits to allow an understanding of their evolution over time. An understanding is a key to allow full insight into the recovery process, and rehabilitation needs of those with TBI and how chronic motor changes post-TBI could provide a novel method for identifying risk of neurodegenerative movement disorders.

## Author contributions

FC, IC, and LC-P contributed to conception and design of the study, reviewed articles for inclusion, contributed to the results, and wrote sections of the manuscript. IC performed the search and organized the database. All authors contributed to manuscript revision, read, and approved the submitted version.
