# Supplementary material for: Chronic motor performance following different traumatic brain injury severity—A systematic review
Source: Front Neurol. 2023 May 11;14:1180353. doi: 10.3389/fneur.2023.1180353 (PMC10243142; doi:10.3389/fneur.2023.1180353)

**Supplementary Tables**

| **Supplementary Table 1. Data extraction table template** | | | | | | | | | | | |
| --- | --- | --- | --- | --- | --- | --- | --- | --- | --- | --- | --- |
| ***Preclinical*** | | | | | | | | | | | |
| **Author** | **Year** | **Title** | **Aim** | **Animal model** | **Sample size** | **TBI severity** | **Injury model and method** | **Time post injury** | **Motor function assessed** | **Result outcome** | |
| Adkins,D.L et al | 2015 | Combining Multiple Types of Motor Rehalibiltation Enhances Skilled Forelimb Use Following Experimental Traumatic Brain injury in Rats | The current study was designed to examine the effects of individual and combined rehabilitative approaches, previously shown to be beneficial following stroke, in an animal model of moderate/severe TBI, the controlled cortical impact (CCI). | All male Hooded long evans rat  3 months old | Moderate TBI and Sham | Moderate-Severe | Controlled cortical injury  4-mm- diameter craniotomy 0.5 mm anterior and 4 mm lateral to the bregma, directly over the FL-SMC.Pneumatic piston cylinder with a 40-mm, angle 18 degree. It (3mm in diameter impactor flat tip) was exposed brain at 0.3 m/sec at a depth of 2.3mm below the cortical surface for 250 msec. | up to 42 days post injury | Foot fault test, | [Result excluded]  Did not compare TBI-untrained to sham individually | |
|  |  |  |  |  |  |  |  |  | single pellet reaching, | CCI-untrained group was significantly different from sham on day 42 (P = .0001) | |
|  |  |  |  |  |  |  |  |  | cylinder test | [Result excluded]  Did not compare CCI-untrained to sham individually | |
| ***Clinical*** | | | | | | | | | | | |
| **Author** | **Year** | **Title** | **Aim** | **Source of population** | **Age range and gender** | **Sample size** | **TBI severity (GCS/ LOC/ PTA/Other measurement)** | **Time post injury** | **Motor function assessed** | **Method used** | **Result outcome** |
| Arce, F.I. et al | 2004 | The scaling of postural adjustments during bimanual load-lifting in traumatic brain injured adults | To examine whether or not patients with TBI are able to scale adequately their postural adjustments during the performance of the dynamic task of bimanual load-lifting. | Unclear | 22-38y/o  All male | TBI=7; Control=10 | Severe (4 subjects with Right hemiparesis; 3 subjects with Left hemiparesis) | 2- more than 10 years | Postural sway | Postural control task: weight shifting | Group differences were found in the magnitude of forward weight shift but not in the relative increase of life postural adjestment(LPA) and post-lift posturaladjustment (PLPA).TBI group demonstrated greater instability during quiet stance phase and limbs heel. |

| **Supplementary Table 2. Literature stated no TBI severity** | | | | | | | | |
| --- | --- | --- | --- | --- | --- | --- | --- | --- |
| **Author** | **Year** | **Species** | **Method** | **Severity (Based on surgical procedure)** | **Depth and velocity** | **Motor performance** | **Cognitive performance** | **Tissue loss** |
| **Adkins, D.L et al** | 2015 | Rat | CCI | Moderate-Severe | 3m/sec for 300ms | Substantial deficits> 72 hours | na | Overt cortical lesion extending to subcortical tissue  Mean: 10mm^3^ |
| **Arun,P et al** | 2020 | Rat | Blast stimulator | Mild | Peak 19 psi blast | Based on the parameters in study by Beamer *et al.* ^1^ | | |
| **Bajwa, N.M et al** | 2016 | Rat | CCI | Moderate | 5m/sec, 1mm depth 200ms | Substantial deficits < 72h followed by sustained minor deficits | Minor deficits <1 week with complete recovery | Overt cortical lesion extending to subcortical tissue  Mean: 5mm^3^ |
| **Cline M.M et al** | 2017 | Mice | CCI | Severe | 6m/sec, 1mm depth for 200msec | Substantial deficits < 72h followed by sustained minor deficits | Substantial deficits >1 week without  complete recovery | na |
| **Daglas,M et al** | 2019 | Mice | CCI | Moderate | 5m/sec, 2mm depth for 150ms | Did not access <72hr but followed by sustained minor deficits | na | Overt cortical lesion extending to subcortical tissue |
| **Hanscom,M et al** | 2021 | Mice | CCI | Moderate-Severe | 6m/sec, 2mm depth | Substantial deficits >72 h | Substantial deficits >1 week without  complete recovery | na |
| **Hoffman,S et al** | 2003 | Rat | CCI | Moderate-Severe | 2.25m/sec,50msec | Substantial deficits <72 h followed by complete  recovery | Minor deficits >1 week | Overt cortical lesion extending to subcortical tissue 20mm^3^ (medial frontal cortex) |
| **Islam,M et al** | 2021 | Mice | CCI | Severe | 2.5m/sec, 2mm depth | Did not access <72hr but followed by sustained minor deficits | Substantial deficits >1 week without  complete recovery | Overt cortical and hippocampal lesion |
| **Komoltsev et al** | 2021 | Rat | LFP | Severe | 2.4-3.6atm | Based on the parameter of fluid percussion injury | | |
| **Leconte,C. et al** | 2020 | Mice | CCI | Moderate | 3.5m/sec, depth 1mm for 50 sec | Did not access <72h but followed by minor deficits | Substantial deficits >1 week | Overt cortical lesion extending to subcortical tissue |
| **Pruitt,D et al** | 2014 | Rat | CCI | Moderate | 3m/sec, 2mm depth 5sec | Did not access <72hr but followed by sustained minor deficits | na | Overt cortical lesion extending to subcortical tissue |
| **Pruitt.D et al** | 2017 | Rat | CCI | Moderate | 3m/sec, 2mm depth 5sec | Did not access <72hr but followed by sustained minor deficits | na | Overt cortical lesion extending to subcortical tissue |
| **Sabbagh,J.J et al** | 2016 | Mice | Air Canon Blast | Moderate | 50 psi blast | Based on the parameters in study by Beamer *et al.* ^1^ | | |
| **Schonfeld,L.M et al** | 2017 | Rat | CCI | Severe | 3m/sec, 5mm depth | Did not access <72hr, complete recovery after | na | Overt cortical lesion extending to subcortical tissue  20mm^3^ |
| **Scott,T.L and C.Vonder Haar** | 2019 | Rat | CCI | Moderate-Severe | 3m/sec, 2.5mm depth 500msec dwell time | Did not access <72hr, complete recovery after | a complete recovery observed after a week | Overt cortical lesion extending to subcortical tissue 20mm^3^ (frontal injury) |
| **Soblosky,J.S et al** | 1997 | Rat | CCI | Moderate-Severe | 5.2m/sec | Substantial deficits < 72h followed by sustained minor deficits | na | Overt cortical lesion extending to subcortical tissue |
| **Toshkezi,G et al** | 2018 | Mice | CCI | Moderate | 1.5m/sec, 2mm depth, 8.5 sec contact time | Deficits seen at 12 weeks PI | minor  deficits at 7 weeks PI | na |
| **Tan, X.L et al** | 2020 | Mice | FP | Moderate | 1-1.5atm | Based on the parameter of fluid percussion injury | | |
| **Thau-Zuchman et al** | 2021 | Mice | CCI | Severe | 3m/sec, 2.2mm depth 100ms dwell time | Substantial deficits >72 h | Substantial deficits >1 week without  complete recovery | Overt cortical and hippocampal lesion |
| **Vogel,A et al** | 2020 | Mice | CCI | Moderate-Severe | 6m/sec, 1.5mm depth, 200msec dwell time | Did not access <72hr, complete recovery after | na | Overt cortical lesion extending to subcortical tissue |

Text/ boxes highlighted in green are the criteria used to classify TBI severity. If criteria are met in more than one classification, the classification with the highest severity was assigned. Parameters are listed in S. Table 2. CCI=Control cortical impact; FP= Fluid percussion injury; na=not applicable.

| **Supplementary Table 3. Parameters for controlled cortical impact injury** | | | | | |
| --- | --- | --- | --- | --- | --- |
| **Severity** | **Depth** | **Velocity** | **Cognitive performance** | **Motor performance** | **Tissue loss** |
| **Mild** | <=1mm | <4m/sec | Minor deficits <1 week with complete recovery | Minor deficits <24 h | None or confined to cortical layer |
| **Moderate** | >1mm; <=2mm | 4-5m/sec | Substantial deficits <1 week followed by minor  deficits or complete recovery | Substantial deficits <72 h followed by complete  recovery or sustained minor deficits | Overt cortical lesion extending to subcortical tissue |
| **Moderate-Severe** | >2mm; <=3mm | >5m/sec | Substantial deficits >1 week without  complete recovery | Substantial deficits >72 h |  |
| **Severe** | >3mm |  |  |  | Overt cortical and hippocampal lesion |

This table is modified from the review via Siebold *et al.*, 2018^2^

| **Supplementary Table 4. Parameters included in the Neuroscore** |
| --- |

|  |  | Score | Seeking | Exit circle | Righting reflex | Shoulder adduction on tail supsension | Limb  Flexion on tail suspension | Lateral pulsion | Limb placement | Inclined Plane | Hemiplegia | Limb reflex | Pinna reflex | Corneal reflex | Startle reflex | Circling | Round beam balance | Platform balance | Beam walk | Landing test | Tail reflex | Seizures/spasm | Drag test | Activity | Gait |
| --- | --- | --- | --- | --- | --- | --- | --- | --- | --- | --- | --- | --- | --- | --- | --- | --- | --- | --- | --- | --- | --- | --- | --- | --- | --- |
| Laurer | 2001 | 15 |  |  |  |  |  |  |  |  |  |  |  |  |  |  |  |  |  |  |  |  |  |  |  |
| Shear | 2010 | 12 |  |  |  |  |  |  |  |  |  |  |  |  |  |  |  |  |  |  |  |  |  |  |  |
| Mountney | 2010 | 10 |  |  |  |  |  |  |  |  |  |  |  |  |  |  |  |  |  |  |  |  |  |  |  |
| Fehily | 2019 | 22 |  |  |  |  |  |  |  |  |  |  |  |  |  |  |  |  |  |  |  |  |  |  |  |
| Feng | 2021 | 10 |  |  |  |  |  |  |  |  |  |  |  |  |  |  |  |  |  |  |  |  |  |  |  |
| Huynh | 2020 | 22 |  |  |  |  |  |  |  |  |  |  |  |  |  |  |  |  |  |  |  |  |  |  |  |
| Zhang | 2021 | 18 |  |  |  |  |  |  |  |  |  |  |  |  |  |  |  |  |  |  |  |  |  |  |  |
| Daglas | 2019 | 4 |  |  |  |  |  |  |  |  |  |  |  |  |  |  |  |  |  |  |  |  |  |  |  |
| Sell | 2017 | 21 |  |  |  |  |  |  |  |  |  |  |  |  |  |  |  |  |  |  |  |  |  |  |  |
| Wang | 2019 | 7 |  |  |  |  |  |  |  |  |  |  |  |  |  |  |  |  |  |  |  |  |  |  |  |
| Segovia | 2020 | 28 |  |  |  |  |  |  |  |  |  |  |  |  |  |  |  |  |  |  |  |  |  |  |  |
| Nissinen | 2017 | 28 |  |  |  |  |  |  |  |  |  |  |  |  |  |  |  |  |  |  |  |  |  |  |  |
| Thau | 2021 | 18 |  |  |  |  |  |  |  |  |  |  |  |  |  |  |  |  |  |  |  |  |  |  |  |
| Zhang | 2005 | 28 |  |  |  |  |  |  |  |  |  |  |  |  |  |  |  |  |  |  |  |  |  |  |  |
| Pierce | 1998 | 28 |  |  |  |  |  |  |  |  |  |  |  |  |  |  |  |  |  |  |  |  |  |  |  |


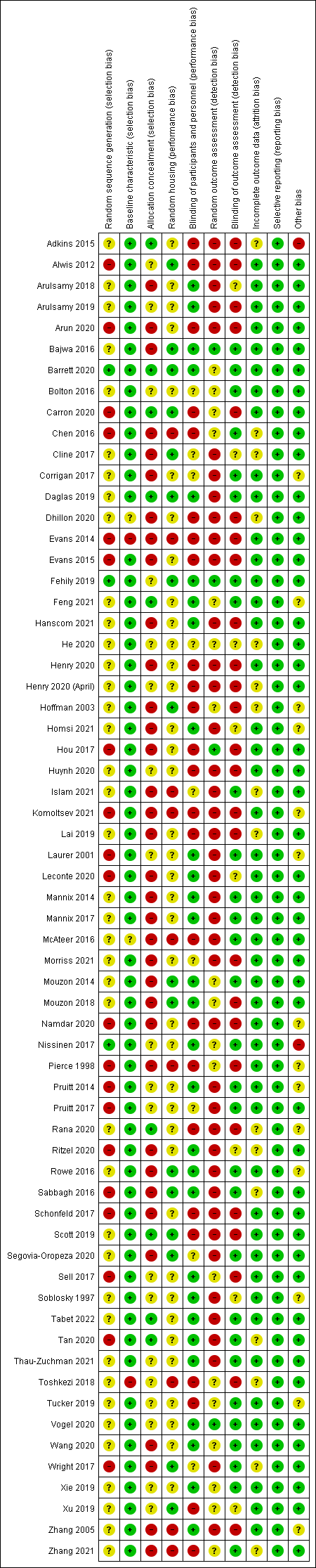

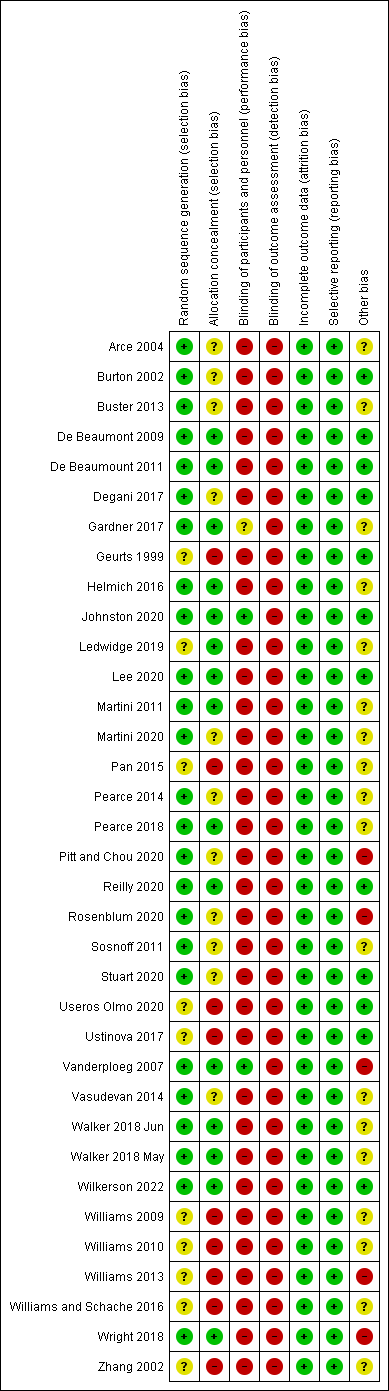


**Supplementary Figure 1. Risk of bias summary.** The authors’ judgement about each risk of bias item for each included study. (a) preclinical studies (b) clinical studies.


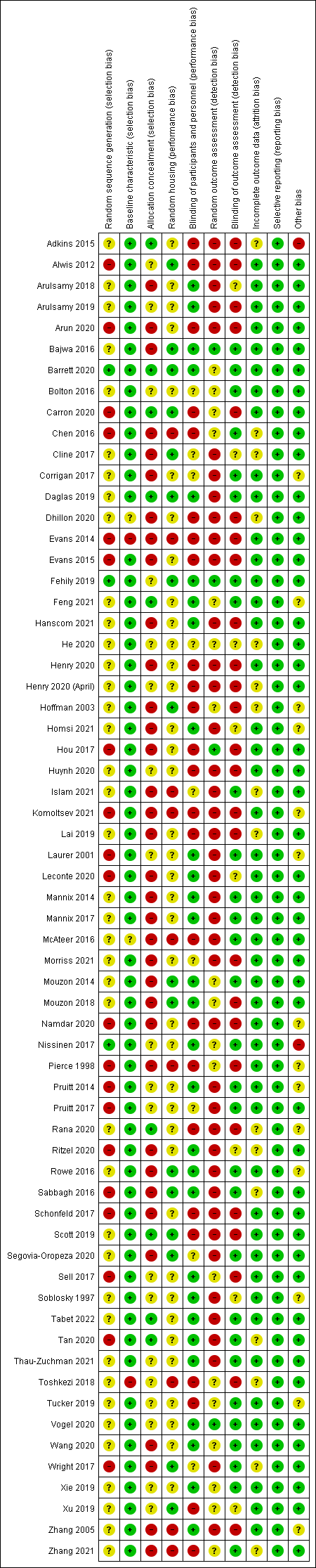

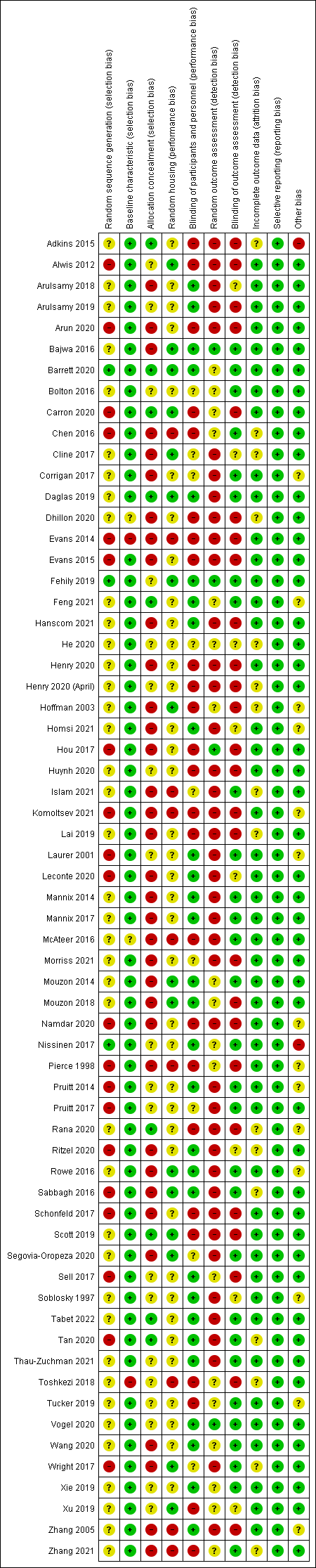

Supplement: Supplementary file 1 [file Data_Sheet_1.docx]
